# Evaluation of Chemical Profile and Biological Properties of Extracts of Different *Origanum vulgare* Cultivars Growing in Poland

**DOI:** 10.3390/ijms25179417

**Published:** 2024-08-30

**Authors:** Izabela Betlej, Natalia Żurek, Tomasz Cebulak, Ireneusz Kapusta, Maciej Balawejder, Anna Kiełtyka-Dadasiewicz, Sławomir Jaworski, Agata Lange, Marta Kutwin, Barbara Krochmal-Marczak, Teresa Kłosińska, Barbara Nasiłowska, Zygmunt Mierczyk, Piotr Borysiuk

**Affiliations:** 1 Institute of Wood Sciences and Furniture, Warsaw University of Life Sciences—SGGW, 159 Nowoursynowska St., 02-776 Warsaw, Poland; teresa_klosinska@sggw.edu.pl; 2 Department of Food Technology and Human Nutrition, Institute of Food Technology and Nutrition, College of Natural Sciences, University of Rzeszów, 4 Zelwerowicza St., 35-601 Rzeszów, Poland; nzurek@ur.edu.pl (N.Ż.); tcebulak@ur.edu.pl (T.C.); ikapusta@ur.edu.pl (I.K.); 3 Department of Chemistry and Food Toxicology, University of Rzeszow, 1a Ćwiklińskiej St., 35-601 Rzeszow, Poland; mbalawejder@ur.edu.pl; 4 Department of Plant Production Technology and Commodity Science, University of Life Sciences in Lublin, Akademicka 15 St., 20-950 Lublin, Poland; anna.kieltyka-dadasiewicz@up.lublin.pl; 5 Garden of Cosmetic Plants and Raw Materials, Research and Science Innovation Center, 4/96 Tarasowa St., 20-819 Lublin, Poland; 6 Department of Nanobiotechnology, Institute of Biology, Warsaw University of Life Sciences, 8 Ciszewskiego St., 02-786 Warsaw, Poland; slawomir_jaworski@sggw.edu.pl (S.J.); agata_lange1@sggw.edu.pl (A.L.); marta_prasek@sggw.edu.pl (M.K.); 7 Department of Plant Production and Food Safety, State University of Applied Sciences in Krosno, 38-400 Krosno, Poland; barbara.marczak@pans.krosno.pl; 8 Institute of Optoelectronics, Military University of Technology, Gen. S. Kaliskiego 2, 00-908 Warsaw, Poland; barbara.nasilowska@wat.edu.pl (B.N.); zygmunt.mierczyk@wat.edu.pl (Z.M.)

**Keywords:** *Origanum vulgare*, polyphenols, antioxidant activity, antimicrobial activity, cytotoxicity

## Abstract

This research studied the phenolic content compared with the antioxidant properties of various *O. vulgare* (Lamiaceae) cultivars grown in Poland. The research results in this paper indicate that the dominant ingredient in all oregano cultivars was rosmarinic acid, known for its strong antioxidant properties. The highest amounts of rosmarinic acid (87.16 ± 4.03 mg/g dm) were identified in the *O. vulgare* spp. hirtum (Link) Ietsw. Other metabolites identified in the studied extracts include luteolin O-di-glucuronide-O-di-pentoside (30.79 ± 0.38 mg/g dm in the ‘Aureum’ cultivar), 4′-O-glucopyranosyl-3′, 4′-dihydroxy benzyl-protocatechuate (19.84 ± 0.60 mg/g dm in the ‘Margerita’ cultivar), and *p*-coumaroyl-triacetyl-hexoside (25.44 ± 0.18 mg/g dm in the ‘Margerita’ cultivar). ‘Hot & spicy’ and ‘Margerita’ cultivars were characterized by the highest activity in eliminating OH^•^ and O_2_^•−^ radicals. Extracts from Greek oregano had the highest ability to scavenge DPPH radicals and chelate iron ions. This research has also provided new evidence that oregano has anti-migratory, cytotoxic properties and influences the viability of gastric cancer cells (the highest cytotoxicity was attributed to the ‘Hot & spicy’ cultivar, which performed the worst in antioxidant properties tests). Extracts from the tested cultivars at a concentration of 0.625% effectively inhibited the growth of *S. aureus* and *P. aeruginosa* bacteria. It seems that the oregano grown in Poland is of good quality and can be successfully grown on a large scale if the appropriate use is found.

## 1. Introduction

*Origanum vulgare* L. is among plants with a broad spectrum of uses. This plant is native to Europe and Central Asia but has escaped gardens and naturalized in parts of the eastern and far western U.S. and Canada. From a botanical point of view, oregano is a perennial plant belonging to the *Lamiaceae*, with a woody base and herbaceous stems that grow up to 20–80 cm. The species is highly variable in its morphological traits and chemical composition. According to the latest taxonomy, six subspecies have been distinguished based on morphological features, i.e., *O. vulgare* L. subsp. glandulosum (Desfontaines) Ietswaart, *O. vulgare* L. subsp. hirtum (Link) Ietswaart, *O. vulgare* L. subsp. gracile (Koch) Ietswaart, *O. vulgare* L. subsp. virens (Hoffmannsegg et Link) Ietswaart, *O. vulgare* L. subsp. vulgare L., and *O. vulgare* L. subsp. viride (Boissier) Hayek [1]. Oregano is a source of substances that give it its characteristic aroma and are responsible for its valuable antioxidant, antibacterial, and anti-inflammatory properties [2,3,4,5,6,7]. The phytochemical composition of the raw material depends on many factors. Both genetic and environmental conditions affect the qualitative and quantitative characteristics of phytoactive substances contained in the plant [8,9] and agronomic factors such as water and nitrogen supply, harvest time, and plant maturity stage [10]. The phytochemical composition of extracts may vary depending on the plant subspecies and habitat type [11].

Chemical substances such as carvacrol, thymol, c-terpinene, and p-cymene contained in the aerial part of oregano are believed to determine its high quality and commercial suitability. Other components in oregano, such as acyclic or bornane-type compounds, significantly influence the raw material features desirable in the pharmaceutical and cosmetic industries [12,13,14]. The properties of oregano raw material and its extracts are widely described in the scientific literature [15,16,17]. Much scientific evidence indicates high antioxidant [17,18] and antimicrobial [19,20] properties of oregano extracts. Other components of oregano that affect the growth of cancer cells are also the subject of research analyses. Chemical compounds contained in oregano seem to affect many mechanisms of cancer cell development. Numerous studies confirm its anticancer effects. Di Liberto et al. [21] have proven that the extract in the form of Sicilian *Origanum vulgare* L. oil induces apoptosis in breast cancer cells (human breast cancer epithelial cell line estrogen (ERα)-positive receptor MCF-7 and triple-negative human breast cancer cell line MDA-MB-231 (ER-, PR-, HER-2-negative). In addition, the anticancer effect of oregano extract on gastric cancer (apoptotic effect on AGS cancer cell lines, reduced ability of cancer cell migration, and thus prevention of cancer cell proliferation) [22], breast adenocarcinoma (reduced viability and proliferation of MCF-7 cancer cells) [23], cervical adenocarcinoma [24,25], and colon adenocarcinoma [26] have been demonstrated. In order to effectively inhibit the development and growth of cancer cells, plant extracts must be as selective as possible about these cells. Studies conducted by Savini et al. [27] on human colon cancer (Caco 2 cell line) show that oregano oil has such properties. Moreover, the entire extract, not a specific component, may be responsible for the observed cytotoxic effects. Studies by Di Liberto et al. [21] on breast cancer cells (MDA-MB-231 and MCF-7 cell lines) also indicate that the cytotoxicity of oregano oil towards cancer cells is mainly due to the combination of its various components. In the presented studies, the effect of oregano extract on the viability of human cancer cells (Mcf-7 breast cancer cell line, Caco-2 c colon cancer cell line, Dld-1, Ht-29, Ls180, U87mg glioblastoma multiforme cell line, U251mg astrocytoma cell line, Sk-mel-28 melanoma cell line, and AGS gastric cancer cell line) and its cytotoxicity towards healthy cells (CCD841CoN colon epithelial cell line) were assessed.

Plant breeders are successively introducing new cultivars and hybrids of known herbal plants to make the offer of ornamental and functional plants at home more attractive. The described cultivars of origanum are valued because of their new attractive appearance (origanum ‘Aureum’) or taste and smell, so they are used as ornamental or spice plants (origanum ‘Hot & spicy’ has a spicy flavor, origanum ‘Margerita’ has a mild taste). Phytochemical substances are responsible for the intensity of the aroma. The same substances also determine the biological properties of the raw material. The preliminary analysis of the composition and properties of *Origanum vulgare* grown in Poland shows a large variability of the composition for individual cultivars [28].

Oregano cultivation in Poland is less widespread than in other southern European countries, but the climatic and soil conditions allow for high yields. Therefore, it seems that oregano grown in Poland could be an alternative to *O. vulgare* varieties grown in regions with a Mediterranean climate. The possibility of growing oregano in climatic conditions other than those in the Mediterranean region and its good antioxidant and antimicrobial properties are essential signals for plant breeders as the demand for this raw material is growing. There are few origanum plantations in Poland, and the raw material is imported from abroad. However, the Joint Research Centre of the European Commission (JRC) published a report in 2021 on the authenticity of herbs and spices that can be purchased in the European Union. After conducting nearly 10,000 analyses, its authors determined that the percentage of samples considered “at risk of adulteration” was 17% for pepper, 14% for cumin, 11% for turmeric, 11% for turmeric, and 11% for saffron. In the case of oregano, this percentage was as high as 48% [29]. Therefore, this work aimed to characterize the polyphenolic composition, antioxidant, and antimicrobial properties of different *O. vulgare* varieties grown in Poland. All these analyses provided a detailed description of the features of *O. vulgare* extracts. They allowed for identifying a variety that may be important for the pharmaceutical and food industries producing functional foods.

## 2. Results

### 2.1. Phytochemical Profile

The identification of the phytochemical composition is presented in Table 1. The analyzed substances were identified based on the spectra of the standards used.

The dominant ingredient in all oregano cultivars was rosmarinic acid. Also, substances such as luteolin O-di-glucuronide-O-di-pentoside, 4′-O-glucopyranosyl-3′, 4′-dihydroxybenzyl-protocatechuate, p-coumaroyl-tri-acetyl-hexoside were present in large amounts in the tested plant raw material. The highest amounts of rosmarinic acid were identified in the *O. vulgare* spp. hirtum (Link) Ietsw (sample O2) (87.16 ± 4.03 mg/g dm). In turn, the *O. vulgare* L. ‘Hot & spicy’ (sample O4) contained the lowest amount of this polyphenol (42.82 ± 1.98 mg/g dm) among all the tested cultivars. The highest amounts of 4′-O-glucopyranosyl-3′ and 4′-dihyroxybenzyl-protocatechuate were identified in *O. vulgare* L. ‘Margerita’ (sample O3). *O. vulgare* L. ‘Aureum’ (sample O5) contained the highest amount of luteolins O-di-glucuronide-O-di-pentoside (30.79 ± 0.38 mg/g dm) among the analyzed oregano cultivars. In turn, salvianolic acid B (Figure S1) dominated in *O. vulgare* spp. hirtum (Link) Ietsw. (sample O2). Moreover, the smallest amounts were determined in *O. vulgare* L. (sample O1). The extract from *O. vulgare* spp. hirtum (Link) Ietsw. (sample O2) contained as much as 40.58 ± 1.31 mg/g dm of compounds determined to be unspecified. Much smaller amounts of unspecified compounds were found in the extracts from ‘Hot & spicy’ (sample O4) and ‘Aureum’ (sample O5). In addition, all cultivars contained small amounts of yunnaneic acid E (Figure S2), characteristic of the genus Sage, which belongs to the same botanical family as oregano, making it highly probable that these compounds occur in both genera. Examples of LC–MS spectra of other substances identified in oregano for the first time are presented in the Supplementary Materials (Figures S3–S5).

### 2.2. Total Phenolic Contents and Antioxidant Activity

The results for the total phenolic content and antioxidant activity of oregano extracts and standards are summarized in Table 2, Tables S1 and S2 (Supplementary Material).

The antioxidant activity of the tested oregano extracts was assessed using seven tests based on different mechanisms of action. The ability of the extracts to remove synthetic ABTS^•+^ and DPPH^•^ radicals, the ability to reduce copper ions (CUPRAC test) and ferric (FRAP test), the chelating potential of metal ions (ChP test), and the ability to scavenge superoxide (O_2_^•−^) and hydroxyl (OH^•^) radicals were assessed. 

Oregano cultivars ‘Hot & spicy’ (sample O4) and ‘Margerita’ (sample O3) were characterized by the highest activity in chelating metal ions and eliminating OH^•^ and O_2_^•−^ radicals. Extracts from the *O. vulgare* spp. hirtum (Link) Ietsw. (O2 sample) had the highest ability to scavenge DPPH radicals and chelate iron ions. Based on the statistical test performed (Table 3), it was shown that there are no statistically significant differences in the ABTS^•+^ radical scavenging activity of extracts from *O. vulgare* spp. hirtum (Link) Ietsw. (sample O2) and *O. vulgare* L. ‘Margerita’ (sample O3).

### 2.3. Cell Viability

The effect of oregano extracts on human cell viability was assessed using the MTS assay. Nine cancer cell lines were selected for analysis, such as breast cancer (Mcf-7), colorectal cancer (Caco-2, Dld-1, Ht-29, Ls180), glioblastoma (U87mg), astrocytoma (U251mg), melanoma (Sk-mel-28) and gastric cancer (AGS). The cytotoxicity of the tested extracts towards healthy colonic epithelial cells (CCD841CoN) was also assessed. The obtained results are summarized in Table 4.

When analyzing individual cancer lines, the Caco-2 line was characterized by the highest sensitivity to the tested extracts. The obtained IC_50_ values ranged from 120.84 μg/mL (sample O4) to 190.15 μg/mL (sample O5). The lowest sensitivity was demonstrated for the Sk-mel-28 line, a malignant melanoma characterized by high aggressiveness and resistance to treatment. Estimated cytotoxicity ranged from 180.49 (sample O4) to 519.34 (sample O1) μg/mL.

However, when analyzing individual oregano species, the highest cytotoxic activity was shown for species 4. Among the nine cancer cell lines, the highest activity was shown against the AGS line (64.66 mg/mL) for the selected species. Therefore, the gastric cancer cell line (AGS) and the fourth oregano species were chosen for further anticancer activity studies.

At the same time, the safety of using the tested extracts was evaluated by assessing their cytotoxic activity towards healthy colonic epithelial cells (CCD841CoN). The values ranged from 256.55 (sample O3) to 558.42 (sample O1) μg/mL. These results were lower compared to the cytotoxic effect estimated for eight cancer cell lines (for O1 oregano), seven cancer cell lines (for O2 oregano), one cancer cell line (sample O3), eight cancer cell lines (for O4 oregano), and three cancer cell lines (for O5 oregano).

The influence of extracts of individual oregano cultivars on the cytotoxicity of cancer cell lines was confirmed by the results of statistical tests (Table 5). It was found that there are no statistically significant differences between the extracts of two oregano cultivars (sample O2 and O3) in terms of the effect on the Dld-1 and Ht-29 cell lines. The lack of statistical significance of the cytotoxic effect on CCd841CoN cell lines was also found between extracts from *O. vulgare* L. ‘Hot & spicy’ (sample O4) and *O. vulgare* L. ‘Aureum’ (sample O5).

Considering the results presented in Table 6, it should be emphasized that the cultivar (O1, O2, O3, O4, O5) has a decisive impact on the tested parameters. The percentage influence of the oregano variety was over 99% in most of the parameters tested. Only in the case of ABTS^•+^ was this impact close to 96%. It is also worth noting that the percentage impact of factors not analyzed in this research was small and amounted to less than 1% (in the case of ABTS^•+^ Error, it was 4.2%).

### 2.4. Cell Migration Potential

**Figure 1:**
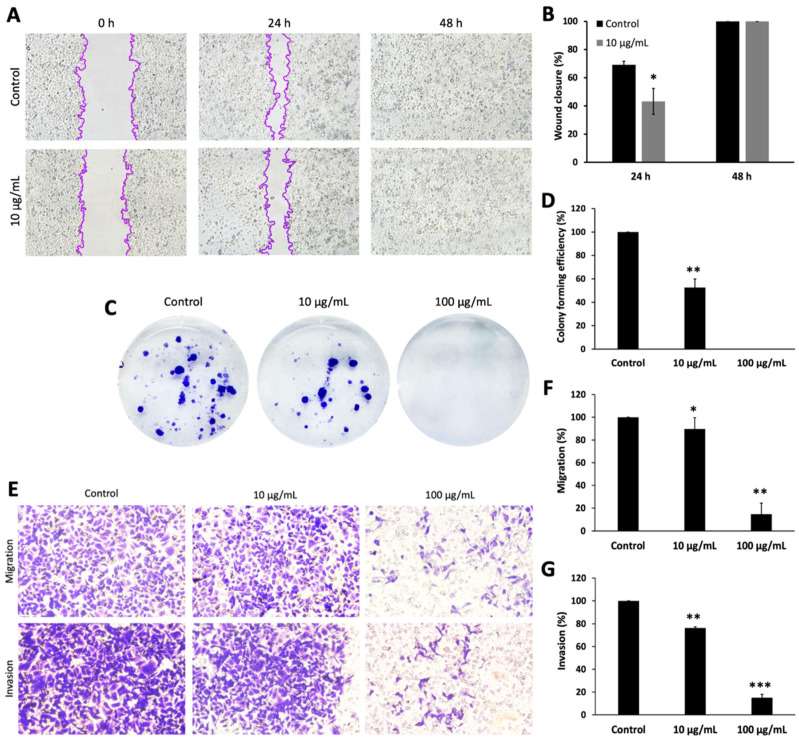
Results of the anticancer activity of the 4th species of oregano extract against AGS cells. (**A**) Representative images of AGS cells in a wound healing assay after treatment with a non-toxic concentration of oregano extract. The percentage of wound closure was assessed 24 and 48 h after scratching. (**B**) Graph showing the percentage of wound closure. It should be noted that 100% means complete closure of the wound. (**C**) Representative images of AGS-stained cells in the clonogenic assay. (**D**) Graph showing the percentage of AGS cell colony formation 14 days after treatment with the tested oregano extract. Untreated cells were used as controls. (**E**) Representative images of AGS-stained cells in the migration and invasion assay. (**F**,**G**) Graphs showing the percentage of inhibition of AGS cell migration and invasion as a result of treatment with oregano extract. Untreated cells were used as controls. All experiments were performed in triplicate. Data are expressed as mean and SD. Significant differences (* < 0.05, ** < 0.01, *** < 0.001) were assessed using the Student’s *t*-test.

In the present study, the migration ability of AGS cells was assessed using two assays: transwell chamber migration and wound healing assay. The results are summarized in Figure 1A,B,E,F, respectively. Treatment with oregano extract (sample O4) at a concentration of 10 mg/mL reduced cell migration through the transwell chamber by 10.32% and by as much as 85.32% at a concentration of 100 mg/mL compared to the control group. Similar observations resulted from the wound healing test, performed at concentrations below the IC_50_ value. In this test, the extract concentration of 10 μg/mL after 24 h of treatment resulted in wound closure by 43.24%, and after 48 h, both treated and untreated cells achieved complete wound closure. Overall, the results obtained for these two tests confirm that oregano extract is an effective agent against the migration of gastric cancer cells.

### 2.5. Colony Formation Efficiency

A colony formation assay was performed to evaluate the long-term effect of oregano extract on the clonogenic potential of AGS cells. As shown in Figure 1C,D, oregano extract significantly inhibited the ability of AGS cells to form colonies in a dose-dependent manner (*p* < 0.05). The percentage of cell colony formation after treatment with the extract at a concentration of 10 μg/mL was reduced by 47.39%. In turn, a concentration of 100 μg/mL completely suppressed the ability of single AGS cells to form colonies. The results suggest that oregano extract effectively inhibits the long-term growth of gastric cancer cells.

### 2.6. Invasion Potential of Cells

In the present study, the invasion capacity of AGS cells was assessed using the Matrigel invasion assay. As shown in Figure 1E,G, the treatment of cells with the extract at a concentration of 10 µg/mL resulted in the inhibition of the invasive potential of cells by 23.7%. However, a concentration of 100 μg/mL reduced the invasive potential of cells by as much as 84.98%. These findings suggest that oregano extract significantly reduces the invasive potential of gastric cancer cells.

### 2.7. Assessment of Bacterial and Yeast Cell Viability

**Figure 2:**
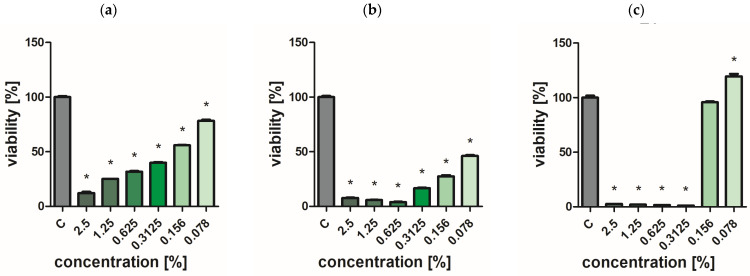
Effect of *O. vulgare* L. (sample O1) on cell viability (**a**) *C. albicans*, (**b**) *P. aeruginosa*, and (**c**) *S. aureus.* Results are presented as a mean ± standard deviation. Statistically significant differences (*p*-value ≤ 0.05) are marked with ‘*’ compared to the control.

**Figure 3:**
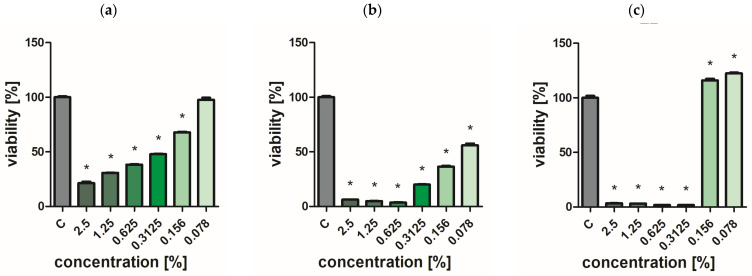
Effect of Greek oregano *O. vulgare* spp. hirtum (Link) Ietsw. (sample O2) on cell viability (**a**) *C. albicans*, (**b**) *P. aeruginosa*, and (**c**) *S. aureus.* Results are presented as a mean ± standard deviation. Statistically significant differences (*p*-value ≤ 0.05) are marked with ‘*’ compared to the control.

**Figure 4:**
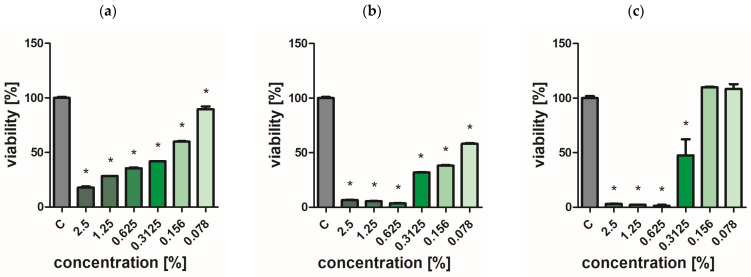
Effect of *O. vulgare* L. ‘Margerita’ (sample O3) on cell viability (**a**) *C. albicans*, (**b**) *P. aeruginosa*, and (**c**) *S. aureus.* Results are presented as a mean ± standard deviation. Statistically significant differences (*p*-value ≤ 0.05) are marked with ‘*’ compared to the control.

**Figure 5:**
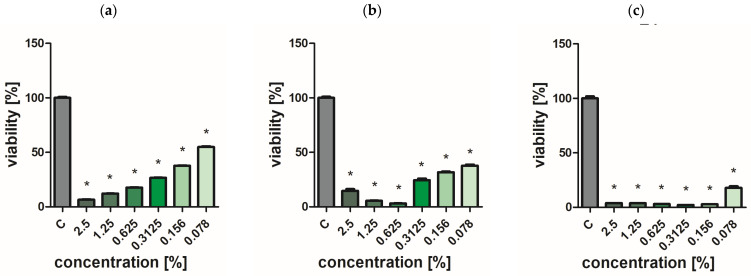
Effect of *O. vulgare* L. ‘Hot & spicy’ (sample O4) on cell viability (**a**) *C. albicans*, (**b**) *P. aeruginosa*, and (**c**) *S. aureus*. Results are presented as a mean ± standard deviation. Statistically significant differences (*p*-value ≤ 0.05) are marked with ‘*’ compared to the control.

**Figure 6:**
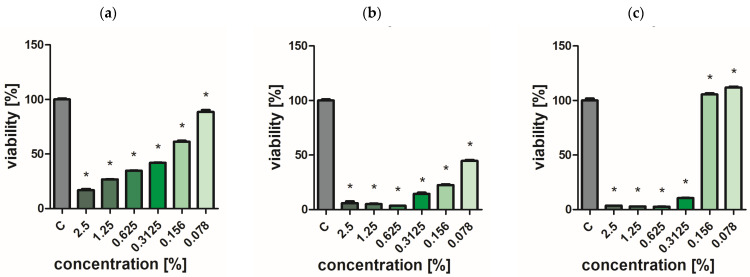
Effect of *O. vulgare* L. ‘Aureum’ (sample O5) on cell viability (**a**) *C. albicans*, (**b**) *P. aeruginosa*, and (**c**) *S. aureus.* Results are presented as a mean ± standard deviation. Statistically significant differences (*p*-value ≤ 0.05) are marked with ‘*’ compared to the control.

Extracts of the analyzed *O. vulgare* cultivars showed weak biocidal activity against yeast-like fungi. Even at the highest concentration used, no effect was achieved to completely inhibit the growth of *C. albicans* (Figure 2, Figure 3, Figure 4, Figure 5 and Figure 6a). In turn, *P. aeruginosa* and *S. aureus* bacteria were sensitive to the extracts. The concentration of extracts above 0.625% significantly reduced the viability of bacterial cells (Figure 2, Figure 3, Figure 4, Figure 5 and Figure 6b,c). The most effective bactericidal activity was observed among the assessed extracts for *O. vulgare* ‘Hot & spicy’ (sample O4) (Figure 5b,c). The weakest effect on bacterial viability was demonstrated by extracts from *O. vulgare* L. ‘Margerita’ (O3 sample) (Figure 4b,c).

## 3. Discussion

Extracts from the tested oregano cultivars show antioxidant properties at the level of 1007.62–1143.18 [µmol TE/g] in the reduction of ABTS^•+^ and 1167.76–2156.16 [µmol TE/g] in the reduction in the DPPH^•^ radical. Relating the obtained results to the results of other authors [41], it should be stated that suitable antioxidant parameters characterize oregano grown in Poland. The obtained oregano can be a successful alternative to *O. vulgare* cultivated in regions with a Mediterranean climate. Antioxidant activity, verified in numerous tests, including using the DPPH radical, as well as in the FRAP, ChP, and CUPRAC methods, showed that four cultivars: *O. vulgare* L. (sample O1), Greek oregano *O. vulgare* spp. hirtum (Link) Ietsw. (sample O2), *O. vulgare* L. ‘Margerita’ (sample O3), and *O. vulgare* L. ‘Aureum’ (sample O5) showed a similar level of antioxidant properties, exceeding the antioxidant activity of the *O. vulgare* L. ‘Hot & spicy’ (sample O4). Total phenolic content analysis indicated that Greek oregano (sample O2) and the ‘Margerita’ (sample O3) cultivars had the highest polyphenol content. Large amounts of polyphenols were identified in the raw materials of these cultivars, such as isosalvianolic acid B and sagerinic acid, which were also present in the remaining cultivars but in smaller amounts. Studies by other authors indicate that these substances have been identified in other oregano species, such as *O. majorana* [42,43]. Among the identified polyphenols, the indicated two—sagerinic acid and yunnaneic acid E—have not been identified in *O. vulgare*. At the same time, it should be added that their identification was based on identical compounds, the presence of which was confirmed in Sage species, which belongs to the same botanical family as oregano [39]. There is a high probability that the newly identified polyphenols may also occur in the characterized oregano genus. The dominant polyphenolic compounds in all oregano cultivars were rosmarinic acid, 4′-O-glucopyranosyl-3′, 4′-dihyroxybenzyl-protocatechuate, and luteolin O-di-glucuronide-O-di-pentoside, which is also confirmed by the work of other researchers [44,45]. Sarrou et al. [46] indicated that rosmarinic acid is the main phenolic acid found in the raw material of *O. vulgare* species. Yfanti et al. [47] found that oregano is only sometimes a raw material rich in rosmarinic acid. However, many factors, e.g., agronomic or genetic, influence the phytochemical composition of the raw material [48].

Polyphenols containing at least one phenolic hydroxyl group in their structure may have antioxidant activity—one of the mechanisms of cell protection against oxidative stress. In the conducted research, *O. vulgare* L. (sample O1) and Greek oregano *O. vulgare* spp. hirtum (Link) Ietsw. were characterized by an exceptionally high ability to engage in radical scavenging activity (sample O2). Since these cultivars show significant differences in the quantitative composition of polyphenols, the ability of these cultivars to have such high radical scavenging activity may be related to the activity of other substances that were not determined in the tests. The antioxidant potential of oregano extracts should also be considered, taking into account the characteristics of the oil substances, which is confirmed by the work of other researchers [49,50].

Studies on cancer cell lines have shown that the best cytotoxicity was characteristic of *O. vulgare* L. extracts ‘Hot & spicy’ (sample O4). This cultivar was characterized by the best results in free radical scavenging tests and the ChP test. At the same time, the amount of identified polyphenols in extracts from this oregano cultivar was the lowest compared to other extracts. Dawra et al. [51] indicate that the antiproliferative effect of oregano extracts on cancer cell lines may be related to the presence of polyphenols in the extracts, especially 4-coumaric acid or naringenin. Small amounts of coumaric acid were determined in the tested ‘Hot & spicy’ cultivar, and naringenin was not detected.

The use of cell lines for the assessment of in vitro cytotoxicity allows obtaining basic but important information on the effect of phytochemicals on the sensitivity of cancer cells [52,53]. Nine cancer cell lines were used in the conducted research, with the Caco-2 cell line being the most sensitive to the tested extracts. At the same time, the most significant cytotoxic effect was obtained for oregano extract, the ‘Hot & spicy’ variety, on the gastric cancer cell line. Extracts from the ‘Hot & spicy’ oregano cultivar showed satisfactory results in studies on reducing the invasive potential of gastric cancer cells and in migration and colony formation tests. The 100 g/mL extract dose reduced cell invasiveness by up to 84.98% and decreased migration by up to 85.32% compared to control tests. In the colony formation assay, complete inhibition of single AGS cells from forming colonies was observed at the dose used.

Many studies indicate that *O. vulgare* extracts inhibit the growth of pathogenic microorganisms [54,55,56]. The research showed that extracts from various oregano cultivars strongly limit the cell viability of *Staphylococcus aureus* and *Pseudomonas aeruginosa* bacteria. The ‘Hot & spicy’ cultivar had excellent killing properties against these two species of bacteria. According to Fouromiti et al. [57], oregano extracts can be used as an antimicrobial agent against Gram-positive and Gram-negative bacteria. The antibacterial effect is mainly attributed to oil ingredients such as carvacrol, thymol, and gamma-terpinene [58]. These substances cause the loss of cytoplasmic material from cells by leaking ions and intracellular compounds [59].

In contrast to the results of other researchers [60], extracts from the tested oregano cultivars showed a weak inhibitory effect on the growth of *C. albicans* fungal cells. Souza et al. [61] found that oregano oils could reduce the size of the *C. albicans* inoculum by 99.9%. Also, Walasek-Janusz et al. [62] proved that small amounts of extracts from various oregano cultivars, at 0.06–0.125 mg/mL, are sufficient to inhibit the development of *C. albicans*. The reason for the poor fungicidal properties of the analyzed oregano cultivars requires a deeper analysis. Still, it seems that the concentration of the extract and volatile substances in the extract may be the reason for lower fungicidal values compared to the results achieved by other researchers. Lower fungicidal activity may also depend on the extraction method of biologically active substances. Bhat et al. [63] showed in their research on the influence of oregano extracts on the growth of *C. ablicans* fungi that extracts obtained using the maceration technique were characterized by worse fungicidal activity compared to the essential oil.

## 4. Materials and Methods

### 4.1. Materials and Reagents

Gallic acid, neocuproine, crystal violet, paraformaldehyde, EDTA (ethylenediaminetetraacetic acid disodium salt dihydrate), ferrozine, 2-Deoxy-D-ribose, NBT (nitrotetrazolium blue chloride), NADH (β-Nicotinamide adenine dinucleotide, reduced disodium salt hydrate), PMS (phenazine methosulfate), phosphate-buffered saline (PBS), RPMI-1640 medium, Dulbecco’s Modified Eagle Medium (DMEM), McCoy’s 5A medium, antibiotics (100 U/mL penicillin and streptomycins), fetal bovine serum (FBS), 0.25% trypsin-EDTA, 0.4% trypan blue solution, and Matrigel were purchased from Sigma-Aldrich (Steinheim, Germany). CellTiter 96^®®^ AQueous Cell Proliferation Assay was purchased from Promega (Madison, WI, USA). All other chemicals were purchased from Chempur (Piekary Śląskie, Poland).

### 4.2. Plant Material

The plant material was obtained from five origanum cultivars: common oregano *O. vulgare* L. (sample O1); greek oregano *O. vulgare* spp. hirtum (Link) Ietsw. (sample O2); *O. vulgare* L. ‘Margerita’ (sample O3); *O. vulgare* L. ‘Hot & spicy’ (sample O4); and *O. vulgare* L. ‘Aureum’ (sample O5).

Plants of all cultivars were obtained from the collection of the Garden of Cosmetic Plants and Raw Materials of the Research and Science Innovation Center in Wola Zadybska near Lublin (Poland) (51°44′49″ N 21°50′38″ E). Botanical identification was performed by the Curator of the Department of Aromatic Plants, Dr. Anna Kiełtyka-Dadasiewicz. The plants were grown in clay soil of loess origin. The upper, leafy, flowering shoots were collected from a 2-year-old plantation in June 2022, in the full flowering phase. The samples were dried for 3 h to achieve a constant humidity level of 12%. After drying (at 35 °C), the stems were manually separated and discarded as the least valuable parts of the raw material. The crushed herb was used as a raw material to obtain extracts. Voucher specimens were deposited in the Research and Science Innovation Center.

### 4.3. Preparation of the Extract

Dried oregano was ground, mixed with 50% methanol, and subjected to extraction assisted by ultrasonic waves (30 °C, 20 min, 50 Hz) (Sonic 10, Polsonic, Poland) [64]. After this time, the samples were centrifugated (Centrifuge 5430, Eppendorf, Hamburg, Germany) at 10,000× *g* for 10 min, and received supernatants were pre-evaporated on a rotary evaporator (R-215 Rotavapor System, Buchi, Switzerland) and lyophilized (ALPHA 1–2 LD plus, Osterode, Germany). The obtained powders were dissolved in water and analyzed.

### 4.4. Identification of Phenolic Compounds

Polyphenolic compounds were determined using ultra-performance liquid chromatography (UPLC-PDA-MS/MS) of the Waters ACQUITY system (Waters, Milford, MA, USA). The UPLC system (UPLC-PDA-MS/MS) is equipped with a binary pump manager, column manager, sample manager, a photodiode array detector (PDA), and a tandem quadrupole mass spectrometer (TQD) with an electrospray ionization source (ESI). The separation of polyphenols was performed using a 1.7 µm, 100 mm × 2.1 mm UPLC BEH RP C18 column (Waters, Milford, MA, USA). For separation, the mobile phase consisted of 0.1% formic acid in water, *v/v* (solvent A), and 0.1% formic acid in 40% acetonitrile, *v/v* (solvent B). The flow rate was kept constant at 0.35 mL/min for a total duration of 8 min. The system was run with the following gradient program: from 0 min 5% B, from 0 to 8 min linearly to 100% B, and from 8 to 9.5 min for washing and returning to the initial conditions. The sample injection volume was 5 μL, and the column was maintained at 50 °C. The following TQD parameters were used: cone voltage 30 V, capillary voltage 3500 V, source and desolvation temperatures 120 °C and 350 °C, respectively, and desolvation gas flow rate 800 L/h. Characterization of individual polyphenolic compounds was carried out on the basis of retention time, mass-to-charge ratio, fragment ions, and comparison with data obtained from commercial standards and literature results. The obtained data were processed in the Waters MassLynx v.4.1 (Waters, Milford, MA, USA). The method was validated for parameters such as linearity, accuracy (relative error, RE), limit of detection (LOD), limit of quantification (LOQ), and precision (relative standard deviation, RSD). Quantification was performed by injecting solutions with known concentrations from 0.05 to 5 mg mL^−1^ (R^2^ ≤ 0.999) of the following phenolic compounds as standards: protocatechuic acid, rosmarinic acid, caffeic acid, p-coumaric acid, salvianolic acid B, and Luteolin 7-*O*-glucoside (Extrasynthese, Genay Cedex, France). Stock-standard solutions of polyphenols were prepared using methanol. Six calibrators determined the peak area ratio of each polyphenol to its nominal concentration. The regression equation was obtained using weighted (1/c2) least squares linear regression. LOD was defined as a signal-to-noise (S/N) ratio of 3:1, and LOQ was defined as a signal-to-noise ratio >10. An allowable RE within ± 20% and intra- and inter-day variability were determined using the relative standard deviation values (RSD), which was determined using relative standard deviation (RSD) values that were <3.5% for all compounds analyzed.

### 4.5. Total Phenolic Content

The total phenolic content was assessed according to the method of Gao et al. [65]. Briefly, 2.0 mL of water, 0.2 mL of Folin–Ciocalteu reagent, and 1.0 mL of 20% sodium carbonate solution were added to the tested extracts. After 60 min, the absorbance of 765 nm was measured (UV–VIS spectrometer, Type UV2900, Hitachi, Japan). The results were expressed in mg gallic acid (GAE)/g dm.

### 4.6. Evaluation of Antioxidant Activity

#### 4.6.1. Superoxide (O_2_^•−^) Radical Scavenging Activity Assay

Superoxide radical scavenging activity was assessed according to the method of Robak and Gryglewski [66]. Oregano extracts were mixed with 1.0 mL of 150 µM NBT, 1.0 mL of 468 µM NADH, and 1.0 mL of 60 µM PMS and left for 5 min. The absorbance was measured at 560 nm. The results were expressed as IC_50_ (µg/mL).

#### 4.6.2. Hydroxyl (OH^•^) Radical Scavenging Activity Assay

Hydroxyl radical scavenging activity was assessed according to the method of Halliwell et al. [67]. Oregano extracts were mixed with 0.1 mL of 0.2 mM 2-deoxyribose, 0.1 mL of 1.0 mM iron ammonium sulfate, 0.1 mL of 1.04 mM EDTA, 0.01 mL of 1.0 mM ascorbic acid, 0.01 mL of 0.1 M perhydrol, 1.0 mL of 2.8% trichloroacetic acid, and 0.5 mL of 1% thiobarbituric acid and heated to 100 °C for 15 min. After this time, the samples were cooled to room temperature, and the absorbance was measured at 532 nm. The results were expressed as IC_50_ (µg/mL).

#### 4.6.3. Chelating Potential of Ferrous Ion

The chelating ability of ferrous ions was assessed according to the method of Żurek et al. [68]. Oregano extracts were mixed with 0.4 mL of 0.25 mM ferrozine and 0.2 mL of 0.1 mM iron II sulfate and left for 10 min. The absorbance was measured at 562 nm. The results were expressed as IC_50_ (µg/mL).

#### 4.6.4. ABTS^•+^ Radical Scavenging Activity

The scavenging activity of extracts on ABTS^•+^ radicals was assessed according to the method of Re et al. [69]. Oregano extracts were mixed with 0.03 mL of ABTS^•+^ solution and left for 6 min. The absorbance was measured at 734 nm. The results were expressed as Trolox Equivalent (mmol TE/g dm).

#### 4.6.5. Determination of Copper Ion Reduction

The copper ion reduction was assessed according to the method of Apak et al. [70]. Oregano extracts were mixed with 1.0 mL of 7.5 mM neocuproine, 1.0 mL of 10 mM copper chloride, and 1.0 mL of 1 M acetate buffer and left for 30 min. The absorbance was measured at 450 nm. The results were expressed as Trolox Equivalent (mmol TE/g dm).

#### 4.6.6. DPPH Test

The DPPH (1,1-diphenyl-2-picrylhydrazyl) free radical scavenging activity test was performed according to the modified method of Brand-Williams [71]. Samples of oregano extracts were diluted to an appropriate concentration within the calibration curve. Measurements were made by placing 0.5 mL of the diluted extract into spectrophotometric cuvettes, adding 2 mL of DPPH solution to them, and mixing after 10 min of incubation in the dark. Spectrophotometric measurements were made at a wavelength of λ = 517 nm against 96% methanol. The results were converted to mmol TE/g dm and as IC_50_ (µg/mL).

#### 4.6.7. Determination of Ferric Reducing Power

The iron-reducing antioxidant power (FRAP) level was determined by reducing Fe^2+^ to Fe^3+^ in an acidic medium using a modified method of Benzie and Strain [72]. To prepare the FRAP reagent, 300 mM acetate, pH 3.6 acetic acid buffer, ferric chloride (FeCl_3_·6H_2_O), and 10 mM 4,6-triridyl-triazine (TPTZ) were prepared in 40 mM HCl. All three solutions were freshly mixed together at 10:1:1 (*v*/*v*/*v*). Performing the determination: 3 mL of FRAP reagent solution was added to the collected 0.5 mL samples and mixed thoroughly. After 10 min at 37 °C, the absorption was measured at a wavelength of 593 nm against distilled water. The results were converted into Trolox Equivalents (mmol TE/g dm).

### 4.7. Cell Culture

The human cell lines Mcf-7 (breast cancer), Caco-2, Dld-1, Ht-29, Ls180 (colorectal cancer), U87mg (glioblastoma), U251mg (astrocytoma), Sk-mel-28 (melanoma), AGS (gastric cancer), and CCD841CoN (colon epithelial cells) were used in the analysis. Selected cell lines were cultured in DMEM, RPMI 1640, or McCoy’s 5A medium, which were enriched with 10% FBS and 1% antibiotics. Cells were incubated at 37 °C in a humidified atmosphere under 5% CO_2_ (incubator CB170, Binder, Tuttlingen, Germany).

### 4.8. Cell Viability Assay

Cell viability was assessed using the MTS test, as described in another study by Żurek et al. [73]. Briefly, the analyzed cell lines were seeded in a 96-well plate at a concentration of 8000 cells/well and incubated for 24 h for cell adhesion. The cells were exposed to five concentrations of oregano extracts (10, 100, 250, 500, and 750 µg/mL). After 48 h, the extracts were removed, the cells were washed with PBS, a fresh medium was added, and the MTS assay was performed according to the manufacturer’s instructions (Promega). Absorbance was measured at 490 nm using a microplate reader (SmartReader 96, Accuris Instruments, St. Louis, MO, USA). The results were expressed as the IC_50_ (µg/mL). Cisplatin was used as a standard (Table S3).

### 4.9. Wound Scratch Test

The wound healing test was performed according to the method of Liang et al. [74]. AGS cells were seeded in 24-well plates at a concentration of 400,000 cells/well and cultured until they reached 80–90% confluence. Then, a wound was made using a sterile pipette tip, and the cells were washed with PBS and treated with extracts at a concentration below the IC_50_ value. Cells were photographed using inverted light microscopy (Oxion Inverso, Euromex, Mataró, Spain) until the wound was closed entirely. Results were expressed as % wound closure.

### 4.10. Migration and Invasion Cell Assay

The ability of cells to migrate and invade was assessed using the transwell chamber assay. Cells (20,000 cells/well) were seeded on a 24-well plate with transwell chambers (pore size 8 μm) and treated with the tested extracts. For the invasion assay, the transwell chamber was coated with 25 µL of Matrigel two hours before seeding. After 48 h, the medium was collected, and the cells were washed with PBS, fixed in 4% paraformaldehyde, treated with ethanol, and stained with 0.5% crystal violet. The stained cells were photographed under a microscope at 20× magnification. The results were expressed as % of migrating and invasive cells relative to the control.

### 4.11. Colony Formation Assay

AGS cells were seeded in a 6-well plate at a concentration of 500 cells/well and placed in the incubator for 24 h. After this time, the medium was removed, and the cells were treated with oregano extract for 48 h. The extracts were then removed, fresh medium was added, and culture was continued for 14 days, changing the medium every 3/4 days. The formed colonies were fixed with methanol, stained with 0.5% crystal violet, and counted under an inverted microscope. Results are expressed as % colony formation relative to control.

### 4.12. Evaluation of Antimicrobial Properties

Antimicrobial properties were assessed using the PrestoBlue viability assay (Cat. No. A13261, Invitrogen, Waltham, MA, USA). The microorganism strains used in the study were *Staphylococcus aureus* (ATCC 25923), *Pseudomonas aeruginosa* (ATCC 27853), and *Candida albicans* (ATCC 90028). Microorganism cultures were diluted in sterile buffer saline to adjust the optical density to 0.5 on the McFarland scale. A total of 90 μL of each microorganism suspension was added into 96-well plates, and 10 μL of each extract was added to final concentrations [%] as follows: 2.5; 1.25; 0.625; 0.3125; 0.156; 0.078. Wells with microorganisms with the addition of 10 μL sterile buffer saline to replace extracts were control samples. Plates were incubated for 24 h at 37 °C. After that, 10 μL of PrestoBlue reagent was added into each well and incubated for 10 min at 37 °C protected from the light. After the incubation, fluorescence was measured (λex = 570 nm, λem = 600 nm) using a microplate reader (ELISA) (Infinite M200, Tecan, Durham, NC, USA). The results were repeated a minimum of three times for each group. Results were calculated as a percentage of the control samples for each microorganism strain. Results are presented as mean values ± standard deviation.

### 4.13. Statistical Analysis

For the PrestoBlue test, a one-way analysis of variance with Tukey’s posthoc test (HSD) was performed using GraphPad Prism 9 software (version 9.2.0, San Diego, CA, USA). The statistically significant differences were considered at *p*-value ≤ 0.05.

Statistical analysis of the results was performed in Statistica version 13 (TIBCO Software Inc., Palo Alto, CA, USA). Analysis of variance (ANOVA) was used to test (α = 0.05) significant differences between oregano cultivars. A comparison of means was performed using the Tukey test, where α = 0.05.

## 5. Conclusions

The tested oregano cultivars are an essential source of antioxidants. In particular, the cultivars *O. vulgare* L. (sample O1) and Greek oregano *O. vulgare* spp. hirtum (Link) Ietsw (sample O2) were characterized by high antioxidant potential, so they could be successfully used where protection against oxidative processes is required; it is worth including these varieties in the daily diet or as food additives. Oregano is also a raw material rich in antioxidant phytochemicals that maintain health properly.

The most important conclusions from the conducted studies are:

- the dominant polyphenol in all oregano extracts tested was rosmarinic acid. The largest amounts were identified in extracts from the *O. vulgare* spp. hirtum (Link) Ietsw. (sample O2), and the smallest amounts in *O. vulgare* L. ‘Hot & spicy’ extracts (sample O4);
- polyphenols such as sagerinic acid and yunnaneic acid E were identified in the extracts, which had not been identified in *O. vulgare* before;
- the prepared extracts show similar antioxidant activity determined in the ABTS^•+^, FRAP, DPPH, and CUPRAC tests;
- the most effective in O_2_^•−^ and OH^•^ radical scavenging activity was the extract the ‘Hot & spicy’ cultivar (sample O4);
- extracts from the ‘Hot & spicy’ cultivar (sample O4) were characterized by the best cytotoxic potential with respect to selected cancer cell lines;
- oregano extracts, especially from the ‘Hot & spicy’ cultivar, have an inhibitory effect on the viability of *S. aureus* and *P. aeruginosa*. At the same time, they show mild fungicidal activity against *C. albicans*.

Therefore, future work should focus on the precise characterization of this oregano variety and the extracts obtained from it. Further detailed studies evaluating the effect of extracts from the ‘Hot & spicy’ cultivar on the cytotoxicity of cancer cell lines should be continued.

At the same time, the characteristics of all oregano cultivars analyzed in this publication require further detailed studies. In particular, a complete quantitative and qualitative characterization of newly identified phenolic compounds is indicated, including their isolation and confirmation of chemical structure using NMR analysis.

It seems that oregano grown in Poland can be successfully used by various industries that use this raw material. At the same time, good antioxidant and antimicrobial properties are essential signals for plant breeders because the demand for this raw material is growing. All the tested cultivars can be grown on a large scale if a suitable application is found.

## Figures and Tables

**Table 1:** Individual phenolic compounds were identified by UPLC-PDA-MS/MS.

No.	Compound	Rt	λ_max_	[M-H] m/z		*Origanum vulgare*					Refs.
								Sample			
						O1	O2	O3	O4	O5	
		min	nm	MS	MS/MS	mg/g dm					
1	Protocatechuic acid *O*-glucoside	1.47	253	315	153	1.68 ± 0.10	1.54 ± 0.09	1.66 ± 0.10	1.15 ± 0.07	1.21 ± 0.07	[30,31,32]
2	3-(3,4-dihydroxyphenyl)-lactic acid	1.88	279	197	179, 135	1.70 ± 0.05	1.92 ± 0.06	2.13 ± 0.07	1.07 ± 0.03	1.56 ± 0.05	[33]
3	Protocatechuic acid	2.07	253, 293	153	109	2.08 ± 0.00	1.32 ± 0.00	9.52 ± 0.02	2.59 ± 0.01	4.23 ± 0.01	[30,31,32]
4	Caffeic acid	3.14	323	179	163, 135	1.85 ± 0.06	1.67 ± 0.05	1.87 ± 0.06	2.38 ± 0.07	2.96 ± 0.09	[30,31,32]
5	Coumaryloquinic acid	3.45	309	337	163	1.87 ± 0.03	1.39 ± 0.02	6.78 ± 0.11	2.53 ± 0.04	1.09 ± 0.02	[34]
6	Luteolin *O*-di-glucuronide	3.63	255, 347	637	285	12.27 ± 0.20	25.57 ± 0.42	16.49 ± 0.27	19.72 ± 0.32	13.41 ± 0.22	[35]
7	*p*-Coumaric acid	3.92	310	163	119	2.31 ± 0.02	2.71 ± 0.03	2.55 ± 0.03	0.87 ± 0.01	1.94 ± 0.02	[30,31,32]
8	Unspecified	4.17	252, 343	537	375	15.49 ± 0.50	40.58 ± 1.31	24.73 ± 0.80	9.78 ± 0.32	9.98 ± 0.32	
9	Luteolin *O*-di-glucuronide-*O*-di-pentoside	4.29	255, 336	799	637, 285	25.08 ± 0.31	11.46 ± 0.14	24.67 ± 0.31	12.84 ± 0.16	30.79 ± 0.38	[30]
10	4′-*O*-glucopyranosyl-3′,4′-dihyroxybenzyl-protocatechuate	4.48	263	437	153	23.28 ± 0.08	15.33 ± 0.05	29.79 ± 0.11	19.71 ± 0.07	27.06 ± 0.10	[36]
11	Luteolin *O*-glucuronide	4.57	255, 346	461	285	4.38 ± 0.16	5.16 ± 0.18	6.28 ± 0.22	3.95 ± 0.14	6.15 ± 0.22	[35]
12	4′-*O*-glucopyranosyl-4′-hydroxybenzyl protocatechuate	4.61	262	421	153	4.24 ± 0.02	4.65 ± 0.02	8.43 ± 0.03	6.54 ± 0.03	8.98 ± 0.04	[37]
13	Salvianolic acid B	4.79	328	717	519, 179	4.76 ± 0.11	19.50 ± 0.46	10.09 ± 0.24	8.47 ± 0.20	9.77 ± 0.23	[38]
14	Isosalvianolic acid B	5.10	327	717	493, 179	6.00 ± 0.20	11.22 ± 0.38	15.96 ± 0.54	6.65 ± 0.23	9.39 ± 0.32	[38]
15	4′-*O*-glucopyranosyl-3′, 4′-dihydroxybenzyl-4-hydroxybenzoate	5.27	258	421	137	12.95 ± 0.39	10.92 ± 0.33	19.84 ± 0.60	4.70 ± 0.14	5.52 ± 0.17	[36]
16	Rosmarinic acid	5.39	327	359	179, 135	63.76 ± 2.95	87.16 ± 4.03	63.21 ± 2.93	42.82 ± 1.98	80.34 ± 3.72	[30,31,32,38]
17	Sagerinic acid	5.46	327	719	359	5.25 ± 0.28	19.20 ± 1.03	14.88 ± 0.80	1.19 ± 0.06	9.03 ± 0.49	[39]
18	*p*-coumaroyl triacetyl-hexoside	5.52	309	451	187, 163	17.99 ± 0.12	13.82 ± 0.10	25.44 ± 0.18	12.52 ± 0.09	9.71 ± 0.07	[38]
19	3-*O*-caffeoyl rosmarinic acid	5.79	329	537	359	3.39 ± 0.03	17.40 ± 0.15	5.45 ± 0.05	1.56 ± 0.01	2.40 ± 0.02	[40]
20	Yunnaneic acid E	5.90	260, 330	571	527, 329	1.48 ± 0.02	0.78 ± 0.01	0.63 ± 0.01	1.23 ± 0.01	2.40 ± 0.03	[39]
21	Salvianolic acid C	6.21	262, 319	491	311	4.66 ± 0.08	12.41 ± 0.22	8.10 ± 0.14	5.53 ± 0.10	7.19 ± 0.13	[39]
22	Salvianolic acid A	6.34	288, 322	493	295, 179	0.70 ± 0.01	1.64 ± 0.02	1.37 ± 0.02	0.45 ± 0.01	3.78 ± 0.04	[39]
23	Salvianolic acid F	7.93	331	313	169, 147	1.14 ± 0.01	0.88 ± 0.01	1.34 ± 0.02	0.88 ± 0.01	2.13 ± 0.03	[39]

Values are expressed as mean and SD from three independent experiments.

**Table 2:** Total phenolic content and antioxidant activity of oregano extracts.

No.	Test	*Origanum vulgare*				
		Sample O1	Sample O2	Sample O3	Sample O4	Sample O5
1	Total phenolic content [mg GAE/g]	258.63 ± 0.57	292.83 ± 0.53	305.71 ± 0.21	246.56 ± 0.43	284.42 ± 1.05
2	ABTS^•+^ [μmol TE/g]	1007.62 ± 17.58	1140.74 ± 5.48	1143.18 ± 4.97	1083.19 ± 22.51	1138.47 ± 5.69
3	DPPH^•^ [μmol TE/g]	2156.16 ± 11.65	1305.28 ± 8.43	1685.40 ± 7.84	11,654.56 ± 4.53	1167.76 ± 15.78
4	FRAP [μmol TE/g]	34.97 ± 0.07	36 ± 0.12	35.71 ± 0.03	32.45 ± 0.14	26.94 ± 0.16
5	CUPRAC [μmol TE/g]	22.97 ± 0.06	25.95 ± 0.11	25.26 ± 0.08	16.69 ± 0.21	20.14 ± 0.03
6	ChP [IC_50_, μg/mL]	340.60 ± 0.68	402.60 ± 0.20	251.62 ± 0.75	163.32 ± 0.77	272.41 ± 0.23
7	O_2_^•−^ radical scavenging activity [IC_50_, μg/mL]	388.44 ± 1.93	403.61 ± 1.87	261.87 ± 1.34	242.63 ± 0.82	384.04 ± 0.92
8	OH^•^ radical scavenging activity [IC_50_, μg/mL]	520.14 ± 3.17	535.43 ± 1.18	300.77 ± 0.55	275.17 ± 0.54	383.92 ± 0.17

Values are expressed as mean and SD from three independent experiments.

**Table 3:** Homogeneous groups regarding the antioxidant properties of extracts from different cultivars of oregano.

	TPC	ABTS^•+^	CUPRAC	ChP	O_2_^•−^	OH^•^
sample O1	b	a	c	d	d	d
sample O2	d	c	d	e	e	e
sample O3	e	c	e	b	b	b
sample O4	a	b	a	a	a	a
sample O5	c	c	b	c	c	c

a–e—homogeneous groups by the Tukey test.

**Table 4:** Effect of oregano on the viability (IC_50_, μg/mL) of ten human cell lines.

No.	Cell Line	*Origanum vulgare*				
		Sample O1	Sample O2	Sample O3	Sample O4	Sample O5
1	Mcf-7	365.75 ± 1.80	361.19 ± 2.32	380.62 ± 2.07	140.96 ± 1.52	318.27 ± 2.42
2	Caco-2	175.41 ± 4.08	133.53 ± 1.89	180.75 ± 1.80	120.84 ± 1.94	190.15 ± 3.58
3	Dld-1	607.65 ± 2.98	594.77 ± 3.87	599.14 ± 2.45	381.97 ± 5.13	534.48 ± 4.54
4	Ht-29	372.82 ± 2.34	300.56 ± 5.89	299.94 ± 6.12	121.69 ± 4.45	170.04 ± 4.99
5	Ls180	353.52 ± 2.33	317.55 ± 2.49	344.10 ± 2.51	260.82 ± 3.69	342.35 ± 3.29
6	U87mg	369.21 ± 1.68	337.83 ± 3.37	308.84 ± 2.59	193.19 ± 1.75	339.26 ± 2.41
7	U251mg	497.82 ± 5.15	298.94 ± 2.07	326.25 ± 1.51	161.31 ± 3.49	305.53 ± 2.25
8	Sk-mel-28	519.34 ± 2.78	395.95 ± 2.57	397.37 ± 1.90	180.49 ± 1.54	513.69 ± 3.57
9	AGS	359.14 ± 2.00	256.20 ± 3.87	310.38 ± 2.37	64.66 ± 0.85	216.82 ± 4.97
10	CCD841CoN	558.42 ± 2.88	370.29 ± 0.94	256.55 ± 3.64	275.60 ± 2.84	273.71 ± 3.29

Values are expressed as mean and SD from 12 independent experiments.

**Table 5:** Homogeneous groups regarding oregano cultivars and their impact on the cytotoxicity of cancer cell lines.

	Dld-1	Ht-29	Ls180	Mcf-7	U87	AGS	Caco-2	Sk-mel-28	u251	CCd841CoN
sample O1	d	d	d	d	d	e	c	d	e	d
sample O2	c	c	b	c	c	c	b	b	b	c
sample O3	c	c	c	e	b	d	d	b	d	a
sample O4	a	a	a	a	a	a	a	a	a	b
sample O5	b	b	c	b	c	b	e	c	c	b

a–e—homogeneous groups by the Tukey test.

**Table 6:** Analysis of variance for the significance of the effect of sample type.

Parameter		*p*-Value	X
TPC	sample	0.033 × 10^−14^	99.946
	Error		0.054
ABTS^•+^	sample	0.074 × 10^−5^	95.818
	Error		4.182
CUPRAC	sample	0.014 × 10^−13^	99.925
	Error		0.075
ChP	sample	0.00 × 10^−16^	99.997
	Error		0.003
O_2_^•−^	sample	0.00 × 10^−16^	99.971
	Error		0.029
OH^•^	sample	0.00 × 10^−16^	99.986
	Error		0.014
Dld-1	sample	0.00 × 10^−16^	99.805
	Error		0.195
Ht-29	sample	0.00 × 10^−16^	99.740
	Error		0.260
Ls180	sample	0.00 × 10^−16^	99.348
	Error		0.652
Mcf-7	sample	0.00 × 10^−16^	99.951
	Error		0.049
U87	sample	0.00 × 10^−16^	99.855
	Error		0.145
AGS	sample	0.00 × 10^−16^	99.909
	Error		0.091
Caco-2	sample	0.00 × 10^−16^	99.048
	Error		0.952
Sk-mel-28	sample	0.00 × 10^−16^	99.960
	Error		0.040
u251	sample	0.00 × 10^−16^	99.920
	Error		0.080
CCd841CoN	sample	0.00 × 10^−16^	99.943
	Error		0.057

*p*—probability of error, X—percentage influence of sample type.

## Data Availability

Data is contained within the article or Supplementary Material.

## Supplementary Materials

The following supporting information can be downloaded at: https://www.mdpi.com/article/10.3390/ijms25179417/s1.

## References

[1] Kosakowska O., Czupa W. (2018). Morphological and Chemical Variability of Common Oregano (*Origanum vulgare* L. subsp. vulgare) Occurring in Eastern Poland. Herba Polonica.

[2] Rojo-Ruvalcaba R.E., García-Cobián T.A., Pascoe-González S., Campos-Bayardo T.I., Guzmán-García L.M., Gil-Gálvez M.C., Escobar-Millán Z., Huerta-García E., García-Iglesias T. (2020). Dose-Dependent Cytotoxicity of the *Origanum vulgare* and Carvacrol on Triple Negative Breast Cancer Cell Line. Proceedings.

[3] Hać-Szymańczuk E., Cegiełka A., Karkos M., Gniewosz M., Piwowarek K. (2018). Evaluation of Antioxidant and Antimicrobial Activity of Oregano (*Origanum vulgare* L.) Preparations During Storage of Low-Pressure Mechanically Separated Meat (BAADER Meat) from Chickens. Food Sci. Biotechnol..

[4] Sakkas H., Papadopoulou C. (2017). Antimicrobial Activity of Basil, Oregano, and Thyme Essential Oils. J. Microbiol. Biotechnol..

[5] Georgantopoulos A., Vougioukas A., Kalousi F.D., Tsialtas I., Psarra A.G. (2023). Comparative Studies on the Anti-Inflammatory and Apoptotic Activities of Four Greek Essential Oils: Involvement in the Regulation of NF-κΒ and Steroid Receptor Signaling. Life.

[6] Shen D., Pan M.H., Wu Q.L., Park C.H., Juliani H.R., Ho C.T., Simon J.E. (2010). LC-MS Method for the Simultaneous Quantitation of the Anti-Inflammatory Constituents in Oregano (Origanum Species). J. Agric. Food Chem..

[7] Lotti C., Ricciardi L., Rainaldi G., Ruta C., Tarraf W., De Mastro G. (2019). Morphological, Biochemical, and Molecular Analysis of *Origanum vulgare* L.. Open Agric. J..

[8] Tuttolomondo T., La Bella S., Leto C., Bonsangue G., Leone R., Gennaro M.C., Virga G., Inguanta R., Licata M. (2016). Effects of Plant Density on the Number of Glandular Trichomes and on Yield and Quality of Essential Oils from Oregano. Nat. Prod. Commun..

[9] Virga G., Sabatino L., Licata M., Tuttolomondo T., Leto C., La Bella S. (2020). Effects of Irrigation with Different Sources of Water on Growth, Yield and Essential Oil Compounds in Oregano. Plants.

[10] D’Antuono L., Galletti G., Bocchini P. (2000). Variability of Essential Oil Content and Composition of *Origanum vulgare* L. Populations from a North Mediterranean Area (Liguria Region, Northern Italy). Ann. Bot..

[11] Spréa R.M., Caleja C., Finimundy T.C., Calhelha R.C., Pires T.C.S.P., Amaral J.S., Prieto M.A., Ferreira I.C.F.R., Pereira E., Barros L. (2024). Chemical and Bioactive Evaluation of Essential Oils from Edible and Aromatic Mediterranean Lamiaceae Plants. Molecules.

[12] Al-Kalaldeh J.Z., Abu-Dahab R., Afifi F.U. (2010). Volatile Oil Composition and Antiproliferative Activity of *Laurus nobilis, Origanum syriacum, Origanum vulgare*, and *Salvia triloba* against Human Breast Adenocarcinoma Cells. Nutr. Res..

[13] Han X., Parker T.L. (2017). Anti-Inflammatory, Tissue Remodeling, Immunomodulatory, and Anticancer Activities of Oregano (*Origanum vulgare*) Essential Oil in a Human Skin Disease Model. Biochim. Open.

[14] Moghrovyan A., Sahakyan N., Babayan A., Chichoyan N., Petrosyan M., Trchounian A. (2019). Essential Oil and Ethanol Extract of Oregano (*Origanum vulgare* L.) from Armenian Flora as a Natural Source of Terpenes, Flavonoids and Other Phytochemicals with Antiradical, Antioxidant, Metal Chelating, Tyrosinase Inhibitory and Antibacterial Activity. Curr. Pharm. Des..

[15] Vahedi G., Khosravi A.R., Shokri H., Moosavi Z., Delirezh N., Sharifzadeh A., Barin A., Shahrokh S., Balal A. (2016). Fungicidal Effect of *Origanum vulgare* Essential Oil Against Candida Glabrata and Its Cytotoxicity Against Macrophages. J. HerbMed. Pharmacol..

[16] Wijesundara N.M., Rupasinghe H.P.V. (2018). Essential Oils from *Origanum vulgare* and *Salvia offcinalis* Exhibit Antibacterial and Anti-Biofilm Activities Against Streptococcus Pyogenes. Microb. Pathog..

[17] Stanojević L.P., Stanojević J.S., Cvetković D.J., Ilić D.P. (2016). Antioxidant Activity of Oregano Essential Oil (*Origanum vulgare* L.). Biol. Nyssana..

[18] Sarikurkcu C., Zengin G., Oskay M., Uysal S., Ceylan R., Aktumsek A. (2015). Composition, Antioxidant, Antimicrobial and Enzyme Inhibition Activities of Two *Origanum vulgare* Subspecies (subsp. vulgare and subsp. hirtum) Essential Oils. Ind. Crops Prod..

[19] Idir F., Van Ginneken S., Coppola G.A., Grenier D., Steenackers H.P., Bendali F. (2022). Origanum vulgare Ethanolic Extracts as a Promising Source of Compounds with Antimicrobial, Anti-Biofilm, and Anti-Virulence Activity Against Dental Plaque Bacteria. Front. Microbiol..

[20] Antoniadou M., Rozos G., Vaou N., Zaralis K., Ersanli C., Alexopoulos A., Dadamogia A., Varzakas T., Tzora A., Voidarou C. (2024). Comprehensive Bio-Screening of Phytochemistry and Biological Capacity of Oregano (*Origanum vulgare*) and *Salvia triloba* Extracts Against Oral Cariogenic and Food-Origin Pathogenic Bacteria. Biomolecules.

[21] Di Liberto D., Iacuzzi N., Pratelli G., Porrello A., Maggio A., La Bella S., De Blasio A., Notaro A., D’Anneo A., Emanuele S. (2023). Cytotoxic Effect Induced by Sicilian Oregano Essential Oil in Human Breast Cancer Cells. Cells.

[22] Balusamy S.R., Perumalsamy H., Huq M.A., Balasubramanian B. (2018). Anti-Proliferative Activity of *Origanum vulgare* Inhibited Lipogenesis and Induced Mitochondrial Mediated Apoptosis in Human Stomach Cancer Cell Lines. Biomed. Pharmacother..

[23] Kubatka P., Kello M., Kajo K., Kruzliak P., Výbohová D., Mojžiš J., Adamkov M., Fialová S., Veizerová L., Zulli A. (2017). Oregano Demonstrates Distinct Tumour-Suppressive Effects in the Breast Carcinoma Model. Eur. J. Nutr..

[24] Öke Altuntaş F., Demirtaş İ. (2017). Real-Time Cell Analysis of the Cytotoxicity of *Origanum acutidens* Essential Oil on HT-29 and HeLa Cell Lines. Turk. J. Pharm. Sci..

[25] Berrington D., Lall N. (2012). Anticancer Activity of Certain Herbs and Spices on the Cervical Epithelial Carcinoma (HeLa) Cell Line. Evid. Based Complement Alternat. Med..

[26] Lee S.-O., Ward P., Brownmiller C.R. (2018). Effect of Mexican Oregano on Colon Cancer Cells. FASEB J..

[27] Savini I., Arnone R., Catani M.V., Avigliano L. (2009). *Origanum vulgare* Induces Apoptosis in Human Colon Cancer Caco2 Cells. Nutr. Cancer.

[28] Baj T., Biernasiuk A., Wróbel R., Malm A. (2020). Chemical Composition and in Vitro Activity of *Origanum vulgare* L., *Satureja hortensis* L., *Thymus serpyllum* L. and *Thymus vulgaris* L. Essential Oils Towards Oral Isolates of Candida Albicans and Candida Glabrata. Open Chem..

[29] Results of an EU Wide Coordinated Control Plan to Establish the Prevalence of Fraudulent Practices in the Marketing of Herbs and Spices. https://food.ec.europa.eu/safety/eu-agri-food-fraud-network/eu-coordinated-actions/herbs-and-spices-2019-2021_en.

[30] Proestos C., Komaitis M. (2013). Analysis of Naturally Occurring Phenolic Compounds in Aromatic Plants by RP-HPLC Coupled to Diode Array Detector (DAD) and GC-MS After Silylation. Foods.

[31] Lagouri V., Alexandri G. (2013). Antioxidant Properties of Greek *O. dictamnus* and *R. officinalis* Methanol and Aqueous Extracts—HPLC Determination of Phenolic Acids. Int. J. Food Prop..

[32] Vallverdú-Queralt A., Regueiro J., Alvarenga J.F.R., Martinez-Huelamo M., Leal L.N., Lamuela-Raventos R.M. (2015). Characterization of the Phenolic and Antioxidant Profiles of Selected Culinary Herbs and Spices: Caraway, Turmeric, Dill, Marjoram and Nutmeg. Food Sc. Technol..

[33] Kulisic T., Dragovic-Uzelac V., Milos M. (2006). Antioxidant Activity of Aqueous Tea Infusions Prepared from Oregano, Thyme and Wild Thyme. Food Technol. Biotechnol..

[34] Clifford M.N., Jaganath I.B., Ludwig I.A., Crozier A. (2017). Chlorogenic Acids and the Acyl-Quinic Acids: Discovery, Biosynthesis, Bioavailability and Bioactivity. Nat. Prod. Rep..

[35] Béjaoui A., Boulila A., Sanaa A., Boussaid M., Fernandez X. (2017). Antioxidant Activity and α-amylase Inhibitory Effect of Polyphenolic-Rich Extract from *Origanum glandulosum* desf. J. Food Biochem..

[36] Matsuura H., Chiji H., Asakawa C., Amano M., Yoshihara T., Mizutani J. (2003). DPPH Radical Scavengers from Dried Leaves of Oregano (*Origanum vulgare*). Biosci. Biotechnol. Biochem..

[37] Nakatani N., Kikuzaki H. (1987). A New Antioxidative Glucoside Isolated from Oregano (*Origanum vulgare* L.). Agric. Biol. Chem..

[38] Vujicic M., Nikolic I., Kontogianni V.G., Saksida T., Charisiadis P., Orescanin-Dusic Z., Blagojevic D., Stosic-Grujicic S., Tzakos A.G., Stojanovic I. (2015). Methanolic Extract of *Origanum vulgare* Ameliorates Type 1 Diabetes Through Antioxidant, Anti-Inflammatory and Anti-Apoptotic Activity. Br. J. Nutr..

[39] Jeshvaghani Z.A., Rahimmalek M., Talebi M., Hossein Goli S.A. (2015). Comparison of Total Phenolic Content and Antioxidant Activity in Different Salvia Species Using Three Model Systems. Ind. Crops Prod..

[40] Exarchou V., Nenadis N., Tsimidou M., Gerothanassis I.P., Troganis A., Boskou D. (2002). Antioxidant Activities and Phenolic Composition of Extracts from Greek Oregano, Greek Sage, and Summer Savory. J. Agric. Food Chem..

[41] Bouloumpasi E., Hatzikamari M., Christaki S., Lazaridou A., Chatzopoulou P., Biliaderis C.G., Irakli M. (2024). Assessment of Antioxidant and Antibacterial Potential of Phenolic Extracts from Post-Distillation Solid Residues of Oregano, Rosemary, Sage, Lemon Balm, and Spearmint. Processes.

[42] Wagdy R., Abdel-Kader R.M., El-Khatib A.H., Linscheid M.W., Handoussa H., Hamdi N. (2023). *Origanum majorana* L. Protects Against Neuroinflammation-Mediated Cognitive Impairment: A Phyto-Pharmacological Study. BMC Complement Med. Ther..

[43] Prerna G., Vasudeva N. (2015). *Origanum majorana* L.—Phyto-Pharmacological Review. Indian J. Nat. Prod. Resour..

[44] Gonceariuc M., Muntean M.V., Butnaraş V., Duda M.M., Benea A., Jelezneac T., Vornicu Z., Cotelea L., Botnarenco P. (2021). Quality Variation of the Moldovan *Origanum vulgare* L. ssp. vulgare L. and *Origanum vulgare* L. ssp. hirtum (Link) Ietsw. Varieties in Drought Conditions. Agriculture.

[45] Naquvi K., Ahamad J., Salma A., Ansari S., Najmi A. (2019). A Critical Review on Traditional Uses, Phytochemistry and Pharmacological Uses of Origanum vulgare linn. Int. Res. J. Pharm..

[46] Sarrou E., Tsivelika N., Chatzopoulou P., Tsakalidis G., Menexes G., Mavromatis A. (2017). A Conventional Nreeding of Greek Oregano (*Origanum vulgare* ssp. hirtum) and Development of Improved Cultivars for Yield Potential and Essential Oil Quality. Euphitica.

[47] Yfanti P., Lazaridou P., Boti V., Douma D., Lekka M.E. (2024). Enrichment of Olive Oils with Natural Bioactive Compounds from Aromatic and Medicinal Herbs: Phytochemical Analysis and Antioxidant Potential. Molecules.

[48] Lucas B., Schmiderer C., Novak J. (2013). Phytochemical Diversity of *Origanum vulgare* L. subsp. vulgare (Lamiaceae) from Austria. Biochem. Syst. Ecol..

[49] Shafiee-Hajiabad M., Novak J., Honermeier B. (2016). Content and Composition of Essential Oil of Four *Origanum vulgare* L. Accessions under Reduced and Normal Light Intensity Conditions. J. Appl. Bot. Food Qual..

[50] Oniga I., Puscas C., Silaghi-Dumitrescu R., Olah N.-K., Sevastre B., Marica R., Marcus I., Sevastre-Berghian A.C., Benedec D., Pop C.E. (2018). *Origanum vulgare* ssp. vulgare: Chemical Composition and Biological Studies. Molecules.

[51] Dawra M., Bouajila J., El Beyrouthy M., Taillandier P., Nehme N., El Rayess Y. (2024). Phytochemical Profile, GC-MS Profiling and In Vitro Evaluation of Some Biological Applications of the Extracts of *Origanum syriacum* L. and *Cousinia libanotica* D.C. Plants.

[52] Stojanović N.M., Mitić K.V., Nešić M., Stanković M., Petrović V., Baralić M., Randjelović P.J., Sokolović D., Radulović N. (2024). Oregano (*Origanum vulgare*) Essential Oil and Its Constituents Prevent Rat Kidney Tissue Injury and Inflammation Induced by a High Dose of L-Arginine. Int. J. Mol. Sci..

[53] Taha M., Elazab S.T., Abdelbagi O., Saati A.A., Babateen O., Baokbah T.A.S., Qusty N.F., Mahmoud M.E., Ibrahim M.M., Badawy A.M. (2023). Phytochemical Analysis of *Origanum majorana* L. Extract and Investigation of Its Antioxidant, Anti-Inflammatory and Immunomodulatory Effects Against Experimentally Induced Colitis Downregulating Th17 Cells. J. Ethnopharmacol..

[54] Kolypetri S., Kostoglou D., Nikolaou A., Kourkoutas Y., Giaouris E. (2023). Chemical Composition, Antibacterial and Antibiofilm Actions of Oregano (*Origanum vulgare* subsp. hirtum) Essential Oil Against *Salmonella typhimurium* and *Listeria monocytogenes*. Foods.

[55] de Almeida P., Blanco-Pascual N., Rosolen D., Cisilotto J., Creczynski-Pasa T., Laurindo J. (2022). Antioxidant and Antifungal Properties of Essential Oils of Oregano (*Origanum vulgare*) and Mint (*Mentha arvensis*) Against *Aspergillus flavus* and *Penicillium commune* for Use in Food Preservation. Food Sci. Technol. Camp..

[56] Leyva-López N., Gutiérrez-Grijalva E.P., Vazquez-Olivo G., Heredia J.B. (2017). Essential Oils of Oregano: Biological Activity Beyond Their Antimicrobial Properties. Molecules.

[57] Fournomiti M., Kimbaris A., Mantzourani I., Plessas S., Theodoridou I., Papaemmanouil V., Kapsiotis I., Panopoulou M., Stavropoulou E., Bezirtzoglou E.E. (2015). Antimicrobial Activity of Essential Oils of Cultivated Oregano (*Origanum vulgare*), Sage (*Salvia officinalis*), and Thyme (*Thymus vulgaris*) Against Clinical Isolates of *Escherichia coli*, *Klebsiella oxytoca*, and *Klebsiella pneumoniae*. Microb. Ecol. Health Dis..

[58] Bairamis A., Sotiropoulou N.-S.D., Tsadila C., Tarantilis P., Mossialos D. (2024). Chemical Composition and Antimicrobial Activity of Essential Oils and Hydrosols from Oregano, Sage and Pennyroyal Against Oral Pathogens. Appl. Sci..

[59] Al Kamaly O., Alanazi A.S., Conte R., Imtara H. (2023). Phytochemical Composition and Insight into Antibacterial Potential of Origanum vulgare Essential Oil from Saudi Arabia Using In Vitro and In Silico Approaches. Processes.

[60] Cleff M.B., Meinerz A.R., Xavier M., Schuch L.F., Schuch L.F., Araújo Meireles M.C., Alves Rodrigues M.R., de Mello J.R. (2010). In Vitro Activity of *Origanum vulgare* Essential Oil Against Candida Species. Braz. J. Microbiol..

[61] Souza N.A.B., de Oliveira Lima E., Guedes D.N., de Oliveira Pereira F., de Souza E.L., de Sousa F.B. (2010). Efficacy of Origanum Essential Oils for Inhibition of Potentially Pathogenic Fungi. Braz. J. Pharm. Sci..

[62] Walasek-Janusz M., Grzegorczyk A., Malm A., Nurzyńska-Wierdak R., Zalewski D. (2024). Chemical Composition, and Antioxidant and Antimicrobial Activity of Oregano Essential Oil. Molecules.

[63] Bhat V., Sharma S.M., Shetty V., Shastry C.S., Rao C.V., Shenoy S., Saha S., Balaji S. (2018). Characterization of Herbal Antifungal Agent, *Origanum vulgare* Against Oral *Candida* spp. Isolated from Patients with Candida-Associated Denture Stomatitis: An In Vitro Study. Contemp. Clin. Dent..

[64] Żurek N., Kapusta I., Cebulak T. (2020). Impact of Extraction Conditions on Antioxidant Potential of Extracts of Flowers, Leaves and Fruits of Hawthorn (*Crataegus × macrocarpa* L.). Food Sci. Technol. Qual..

[65] Gao X., Ohlander M., Jeppsson N., Björk L., Trajkovski V. (2000). Changes in Antioxidant Effects and Their Relationship to Phytonutrients in Fruits of Sea Buckthorn (*Hippophae Rhamnoides* L.) during Maturation. J. Agric. Food Chem..

[66] Robak J., Gryglewski R.J. (1988). Flavonoids Are Scavengers of Superoxide Anions. Biochem. Pharmacol..

[67] Halliwell B., Gutteridge J.M.C., Aruoma O.I. (1987). The Deoxyribose Method: A Simple “Test-Tube” Assay for Determination of Rate Constants for Reactions of Hydroxyl Radicals. Anal. Biochem..

[68] Żurek N., Pycia K., Pawłowska A., Kapusta I.T. (2022). Phytochemical Screening and Bioactive Properties of *Juglans regia* L. Pollen. Antioxidants.

[69] Re R., Pellegrini N., Proteggente A., Pannala A., Yang M., Rice-Evans C. (1999). Antioxidant Activity Applying an Improved ABTS Radical Cation Decolorization Assay. Free Radic. Biol. Med..

[70] Apak R., Güçlü K., Özyürek M., Esin Karademir S., Erçağ E. (2006). The Cupric Ion Reducing Antioxidant Capacity and Polyphenolic Content of Some Herbal Teas. Int. J. Food Sci. Nutr..

[71] Brand-Williams W., Cuvelier M.E., Berset C. (1995). Use of a Free Radical Method to Evaluate Antioxidant Activity. LWT–Food Sci. Technol..

[72] Benzie I.F.F., Strain J.J. (1996). The Ferric Reducing Ability of Plasma (FRAP) as a Measure of Antioxidant Power: The FRAP Assay. Anal. Biochem..

[73] Żurek N., Pawłowska A., Kapusta I. (2023). Obtaining Preparations with Increased Content of Bioactive Compounds from Eight Types of Berries. J. Berry Res..

[74] Liang C.-C., Park A.Y., Guan J.-L. (2007). In vitro Scratch Assay: A Convenient and Inexpensive Method for Analysis of Cell Migration in Vitro. Nat. Protoc..

