# Supplementary material for: Evaluation of Chemical Profile and Biological Properties of Extracts of Different Origanum vulgare Cultivars Growing in Poland
Source: Int J Mol Sci. 2024 Aug 30;25(17):9417. doi: 10.3390/ijms25179417 (PMC11395194; doi:10.3390/ijms25179417)
Supplement: Supplementary file 1 [file ijms-25-09417-s001.zip › Supplementary material_19.08a.pdf]

International Journal of
Molecular Sciences

MDPI

Supplementary material

Evaluation of chemical profile and biological propertie of extracts from different *Origanum vulgare* cultivars growing in Poland

Izabela Betlej [1,*], Natalia Żurek [2], Tomasz Cebulak [2], Ireneusz Kapusta [2], Maciej Balawejder [3], Anna Kiełtyka-Dadasiewicz [4], Sławomir Jaworski [5], Agata Lange [5], Marta Kutwin [5], Barbara Krochmal-Marczak [6], Teresa Kłosinska [1], Barbara Nasiłowska [7], Zygmunt Mierczyk [7] and Piotr Borysiuk [1,*]

[1] Institute of Wood Sciences and Furniture, Warsaw University of Life Sciences—SGGW, 159 Nowoursynowska St., 02-776 Warsaw, Poland; piotr_borysiuk@sggw.edu.pl (P.B.); izabela_betlej@sggw.edu.pl (I.B.); teresa_klosinska@sggw.edu.pl (T.K.)

[2] Department of Food Technology and Human Nutrition, Institute of Food Technology and Nutrition, College of Natural Sciences, University of Rzeszów, 4 Zelwerowicza St.; 35-601 Rzeszów, Poland; tcebulak@ur.edu.pl (T.C.); ikapusta@ur.edu.pl (I.K.); nzurek@ur.edu.pl (N.Z.)

[3] Department of Chemistry and Food Toxicology, University of Rzeszow, 1a Ćwiklińskiej St., 35-601 Rzeszow, Poland; mbalawejder@ur.edu.pl (M.B.)

[4] Department of Plant Production Technology and Commodity Science, University of Life Sciences in Lublin, Akademicka 15 St., 20-950 Lublin, Poland; anna.kieltyka-dadasiewicz@up.lublin.pl (A.K.-D.)

[5] Department of Nanobiotechnology, Institute of Biology, Warsaw University of Life Sciences, 8 Ciszewskiego St., 02-786 Warsaw, Poland; slawomir_jaworski@sggw.edu.pl (S.J.); agata_lange1@sggw.edu.pl (A.L.); marta_prasek@sggw.edu.pl (M.P.)

[6] Department of Plant Production and Food Safety, State University of Applied Sciences in Krosno, 38-400 Krosno, Poland; barbara.marczak@pans.krosno.pl (B.K.-M.)

[7] Institute of Optoelectronics, Military University of Technology, gen. S. Kaliskiego 2, 00-908 Warsaw, Poland; barbara.nasilowska@wat.edu.pl (B.N.) zygmunt.mierczyk@wat.edu.pl (Z.M.)

* Correspondence: piotr_borysiuk@sggw.edu.pl (P.B.); izabela_betlej@sggw.edu.pl (I.B.)

Contents:

Table S1. Antioxidant activities of standards.

Table S2. Antioxidant activity of oregano extracts.

Table S3. Effect of Cisplatin on the viability (IC_{50}, µg/ml) of human cell lines.

Figure S1. LC-MS spectra obtained for Salvianolic acid B identified in *Oregano spp*. For the first time.

Figure S2.. LC-MS spectra obtained for Yunnaneic acid E identified in *Oregano spp*. For the first time.

Figure S3. LC-MS spectra obtained for Sagarenic acid identified in *Oregano spp*. For the first time.

Figure S4. LC-MS spectra obtained for Rosmarinic acid caffeoyl identified in *Oregano spp*. For the first time.

Figure S5. LC-MS spectra obtained for Isosalvianolic B identified in *Oregano spp*. For the first time.

Table S1. Antioxidant activities of standards.

Standard	Antioxidant test							
	**ChP**	**$O_2^{\bullet-}$**	**$OH^{\bullet}$**	**$ABTS^{\bullet+}$**	**$DPPH^{\bullet}$**	**FRAP**	**CUPRAC**	**$DPPH^{\bullet}$**
	IC_{50} (μg/mL)					μmol TE/g		
Ascorbic acid	-	83.50 ± 0.41	-	31.93 ± 0.11	5.09 ± 0.07	74.16 ± 0.17	62.19 ± 0.74	36.03 ± 0.06
EDTA	17.11 ± 0.03	-	-	-		-	-	-
Quercetin	-	-	8.93 ± 0.08	-		-	-	-

Abbreviations: ChP, chelating potential of metal ions; $O_2^{\bullet-}$, superoxide radical scavenging activity assay; $OH^{\bullet}$, hydroxyl radical scavenging activity assay; $ABTS^{\bullet+}$, 2,2′-azino-bis(3-etylobezotiazolino-6-6-kwas sulfonowy); $DPPH^{\bullet}$, 2,2-diphenyl-1-picrylhydrazyl; CUPRAC, copper ion reduction assay; FRAP, iron ion reduction capacity; IC_{50}, half the maximum inhibitory concentration; TE, Trolox equivalent; EDTA, ethylenediaminetetraacetic acid. Ascorbic acid, EDTA, and quercetin were used as positive controls; (-) not tested. Values are expressed as mean ± SD.

Table S2. Antioxidant activity of oregano extracts.

No.	Test	*Origanum vulgare*				
		**sample O1**	**sample O2**	**sample O3**	**sample O4**	**sample O5**
1	$DPPH^{\bullet}$ [IC_{50}, μg/mL]	20.11 ± 0.48	30.27 ± 0.70	27.15 ± 0.36	35.84 ± 0.69	37.49 ± 0.74

Values are expressed as mean and SD from 3 independent experiments.

Table S3. Effect of Cisplatin on the viability (IC_{50}, μg/ml) of human cell lines.

No.	Cell line	Cell viability IC_{50}, μg/mL
1	Mcf-7	-
2	Caco-2	1.72 ± 0.11
3	Dld-1	2.58 ± 0.25
4	Ht-29	13.22 ± 0.62
5	Ls180	8.09 ± 0.46
6	U87mg	-
7	U251mg	-
8	Sk-mel-28	-
9	AGS	-
10	CCD841CoN	-

Values are expressed as mean and SD from 8 independent experiments. Abbreviations: (-) not tested.
