# Supplementary figures and images for: Evaluation of Chemical Profile and Biological Properties of Extracts of Different Origanum vulgare Cultivars Growing in Poland
Source: Int J Mol Sci. 2024 Aug 30;25(17):9417. doi: 10.3390/ijms25179417 (PMC11395194; doi:10.3390/ijms25179417)

Origanum\_l 556 (4.737) Cm (556:575)

1: Scan ES-

1.78e6

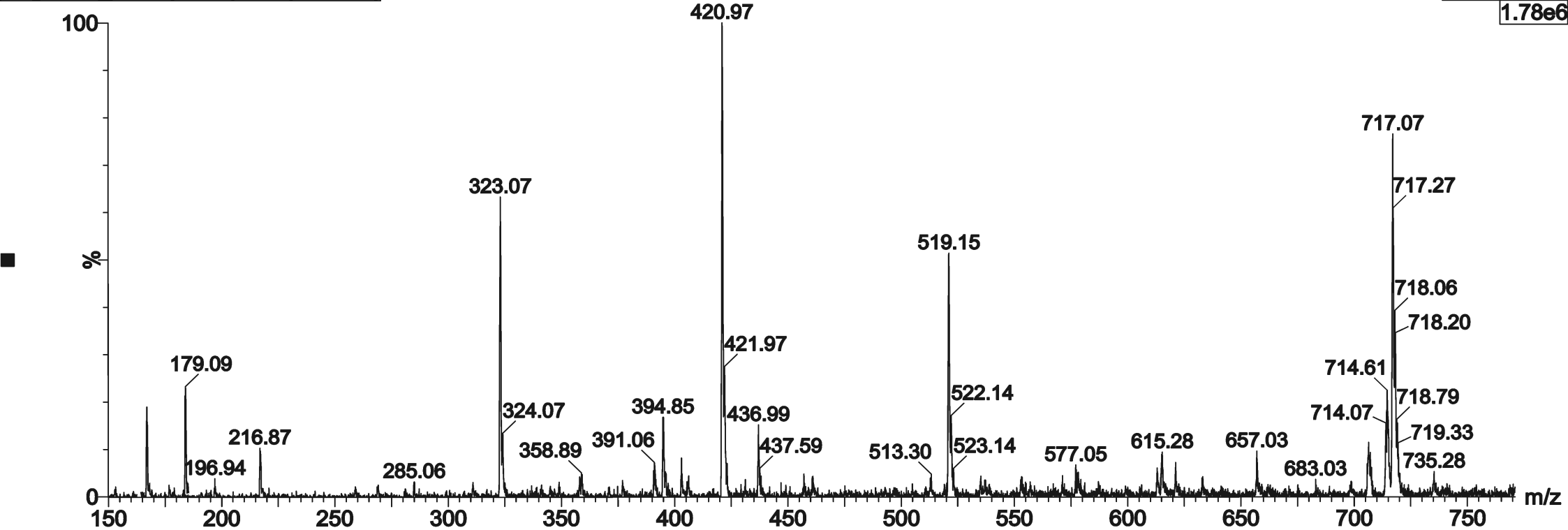

Origanum\_l 5742 (4.784)

2: Diode Array

1.262

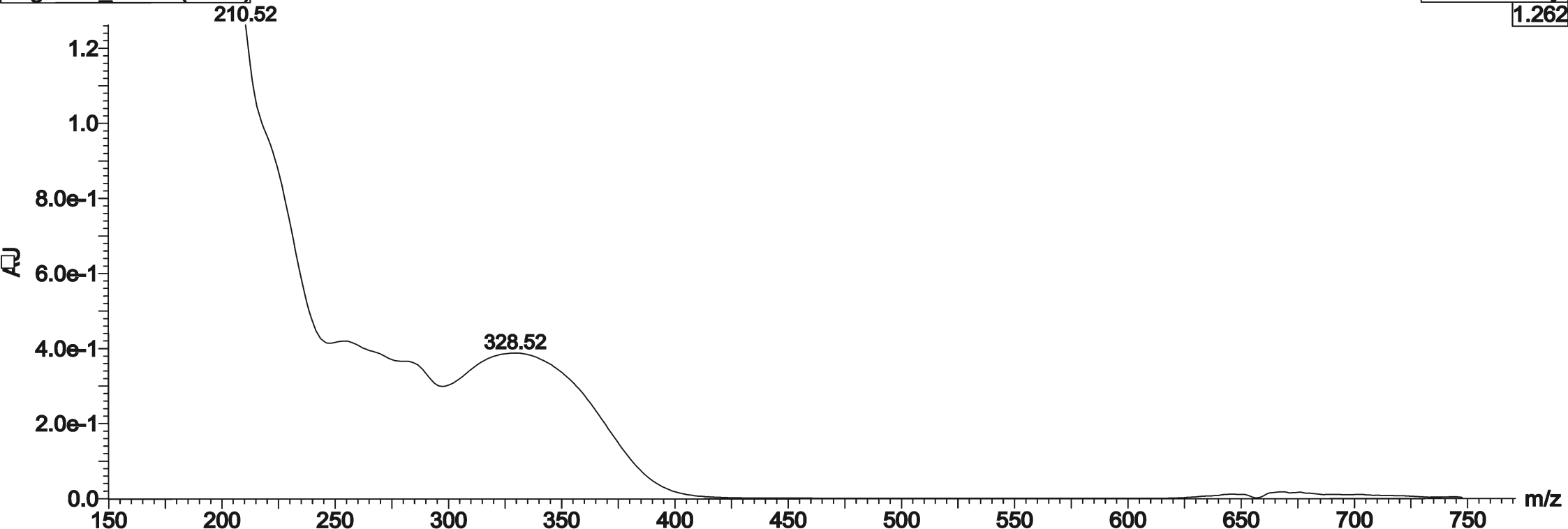

Supplement: Supplementary file 1 [file ijms-25-09417-s001.zip › Figure S1_Compound 13 Salvianolic acid B.pdf]

Origanum\_l 699 (5.956) Cm (691:706)

1: Scan ES-  
3.90e6

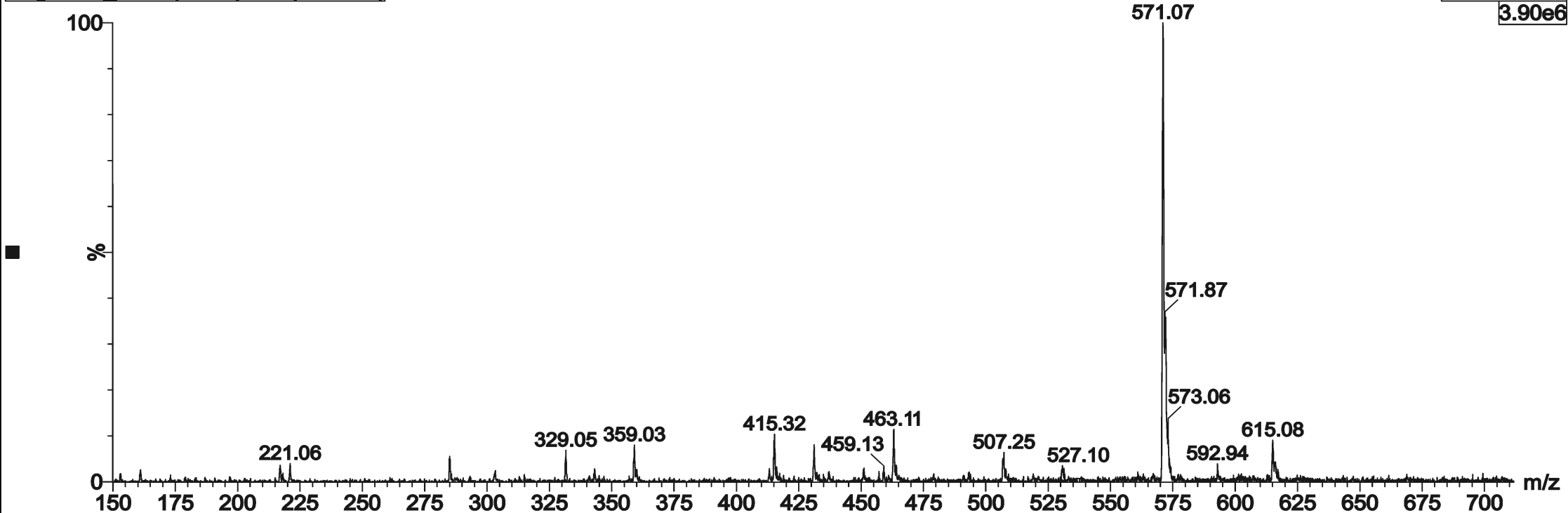

Origanum\_l 7091 (5.909)

2: Diode Array  
1.264

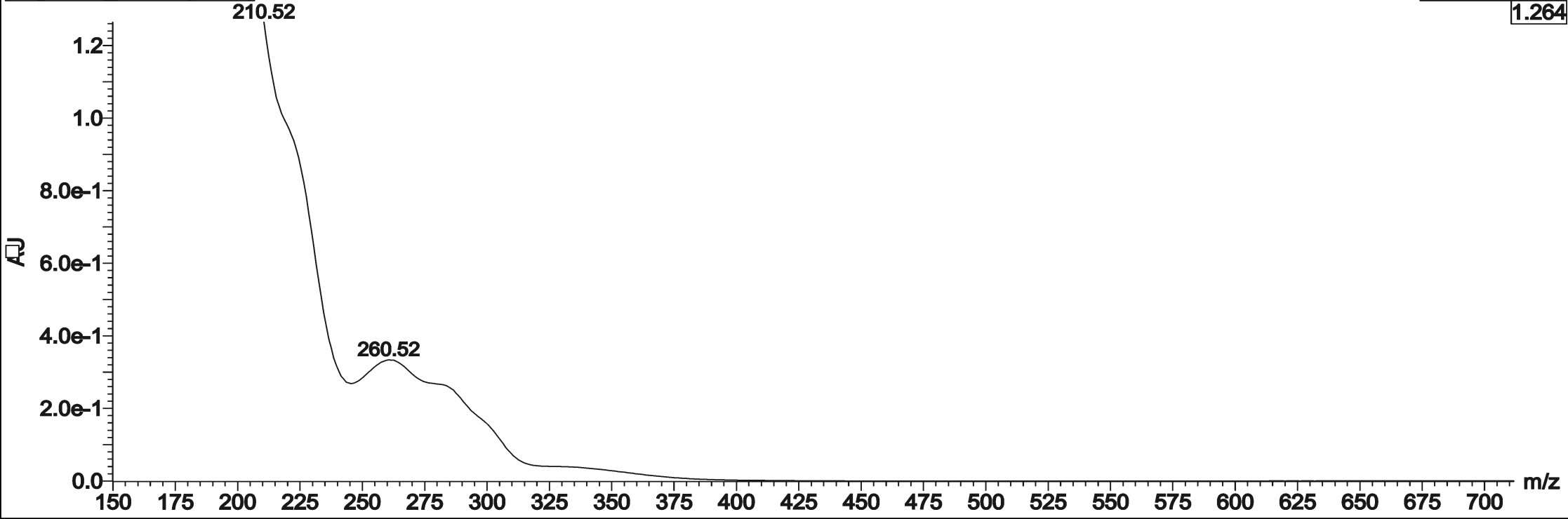

Supplement: Supplementary file 1 [file ijms-25-09417-s001.zip › Figure S2_Compound 20 Yunnaneic acid E.pdf]

Origanum\_l 641 (5.462) Cm (641:657)

1: Scan ES-  
6.13e6

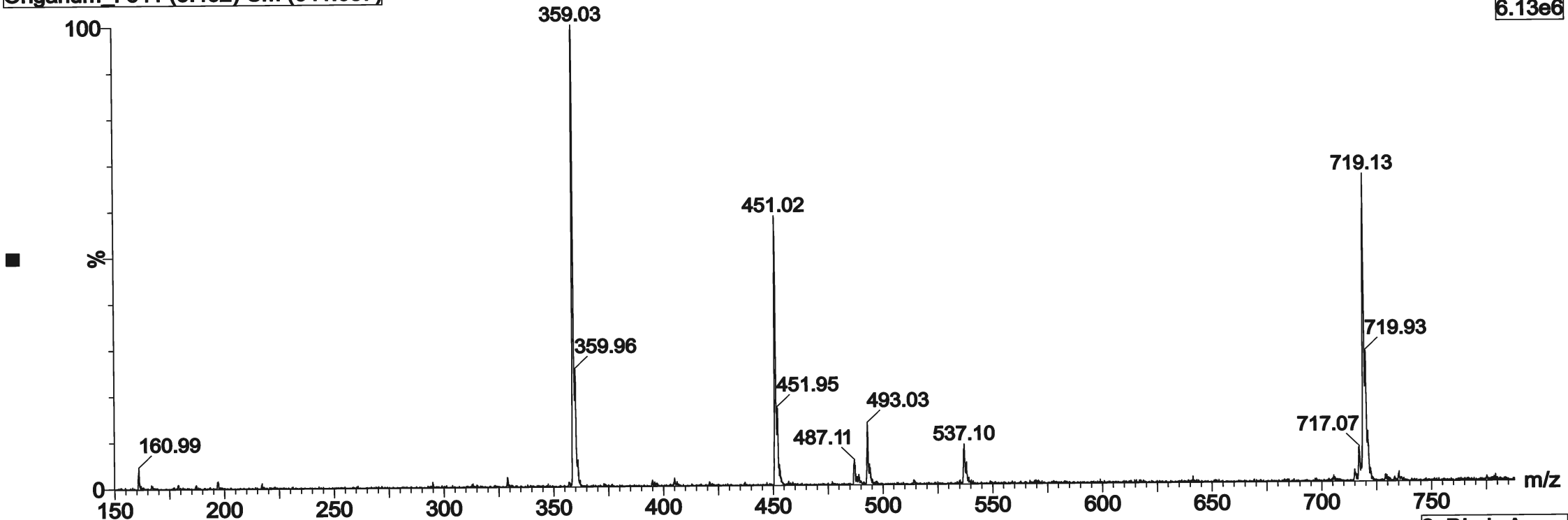

Origanum\_l 6563 (5.469)

2: Diode Array  
2.072

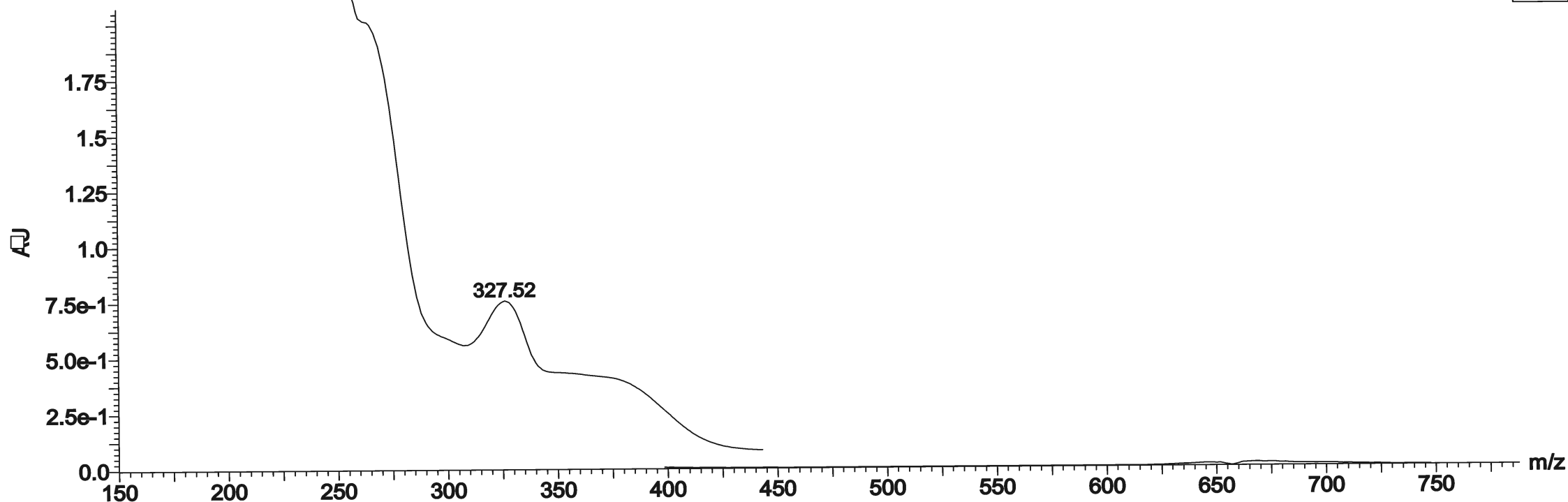

Supplement: Supplementary file 1 [file ijms-25-09417-s001.zip › Figure S3_Compound 17 Sagarenic acid.pdf]

Origanum | 684 (5.828) Cm (671:692)

1: Scan ES-

1.28e6

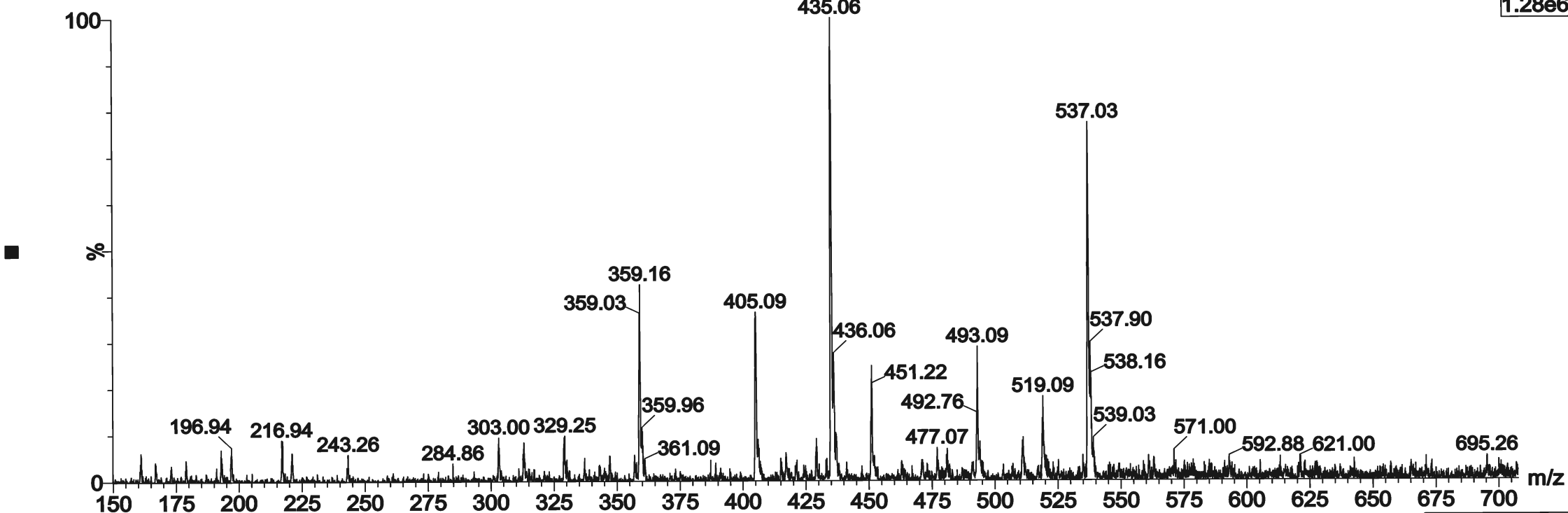

Origanum | 6955 (5.795)

2: Diode Array

1.272

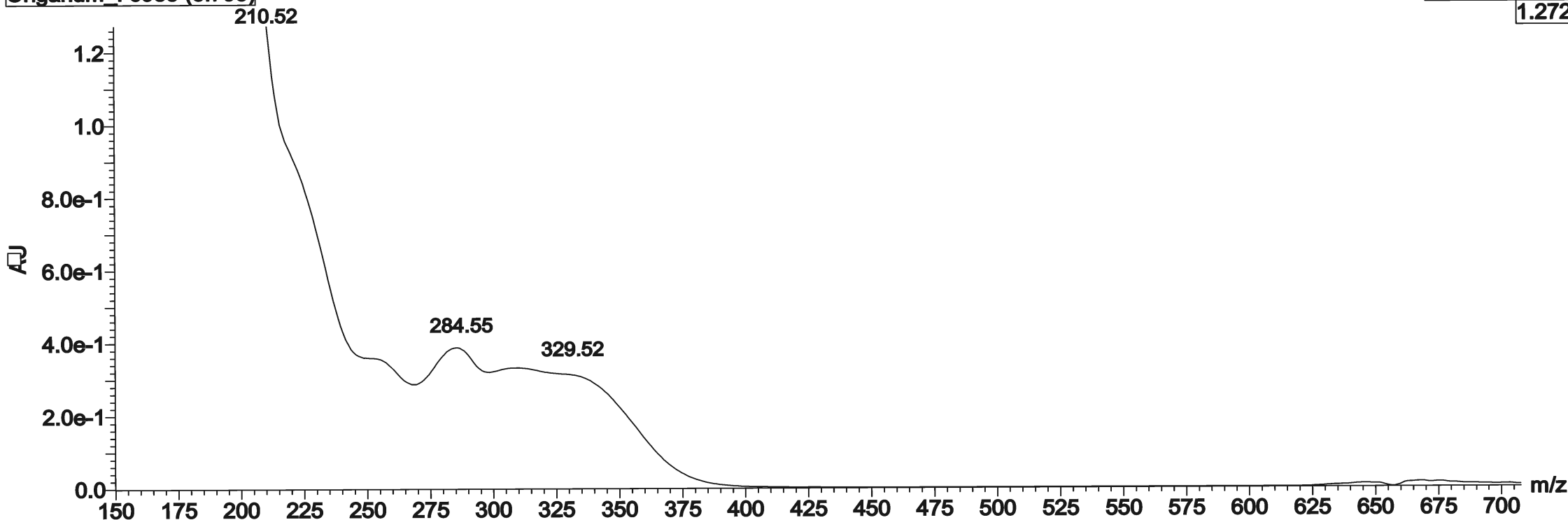

Supplement: Supplementary file 1 [file ijms-25-09417-s001.zip › Figure S4_Compound 19 Rosmarinic acid caffeoyl.pdf]

Origanum\_I 606 (5.163) Cm (601:612)

1: Scan ES-  
2.34e6

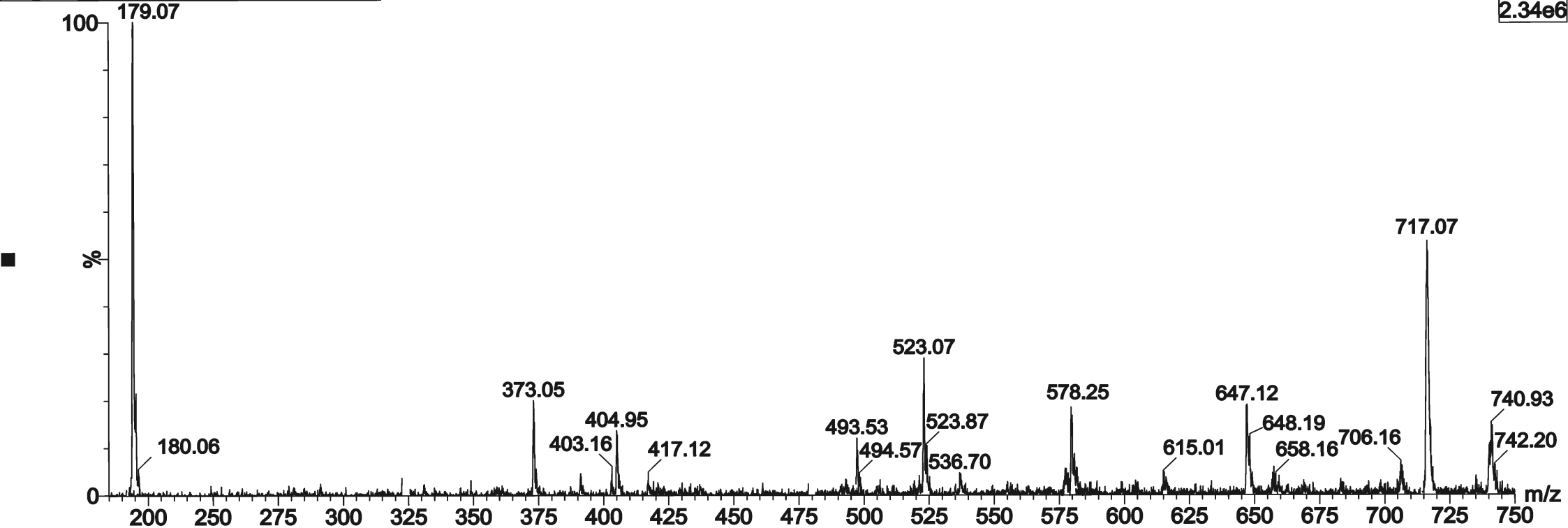

Origanum\_I 6129 (5.107)

2: Diode Array  
1.441

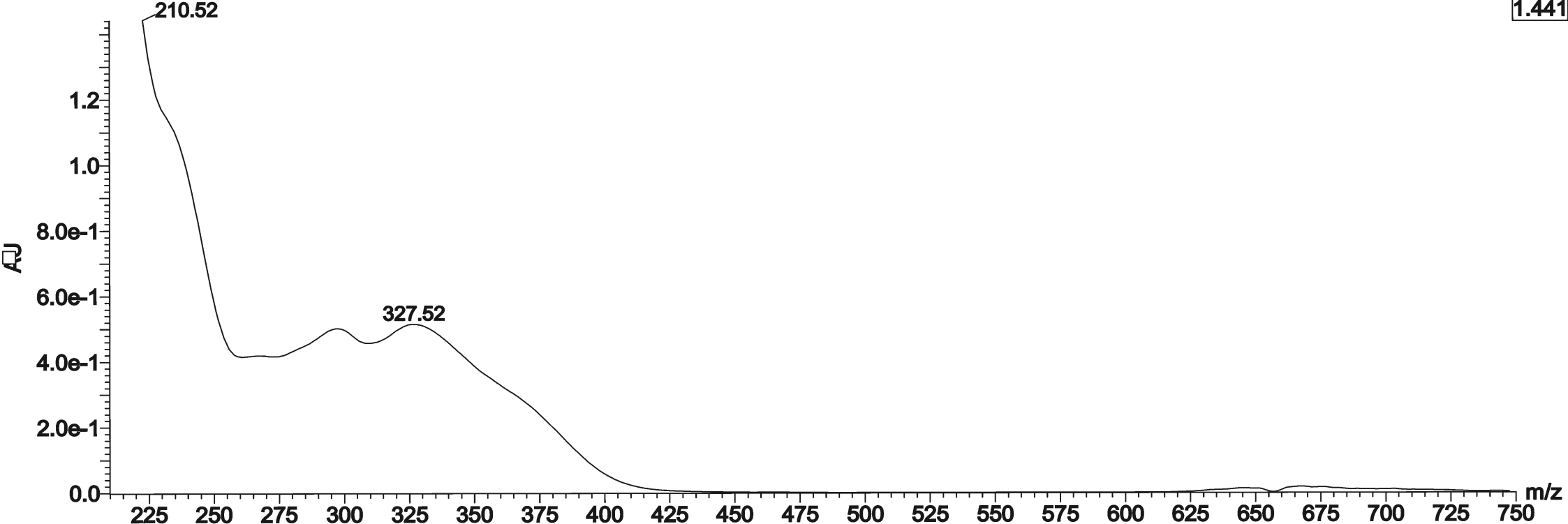

Supplement: Supplementary file 1 [file ijms-25-09417-s001.zip › Figure S5_Compound 14 Isosalvianolic B.pdf]
